# GM1 Gangliosidosis Type II: Results of a 10-Year Prospective Study

**DOI:** 10.1101/2024.01.04.24300778

**Published:** 2024-01-04

**Authors:** Precilla D’Souza, Cristan Farmer, Jean Johnston, Sangwoo T Han, David Adams, Adam L. Hartman, Wadih Zein, Laryssa A. Huryn, Beth Solomon, Kelly King, Christopher Jordan, Jennifer Myles, Elena-Raluca Nicoli, Caroline E Rothermel, Yoliann Mojica Algarin, Reyna Huang, Rachel Quimby, Mosufa Zainab, Sarah Bowden, Anna Crowell, Ashura Buckley, Carmen Brewer, Deborah Regier, Brian Brooks, Eva Baker, Gilbert Vézina, Audrey Thurm, Cynthia J Tifft

**Affiliations:** Office of the Clinical Director and Medical Genetics Branch, National Human Genome Research Institute, 10 Center Drive, Bethesda MD USA; Neurodevelopmental and Behavioral Phenotyping Service, National Institute of Mental Health, 10 Center Drive, Bethesda MD USA; Office of the Clinical Director and Medical Genetics Branch, National Human Genome Research Institute, 10 Center Drive, Bethesda MD USA; Medical Genetics Branch, National Human Genome Research Institute, 10 Center Drive, Bethesda MD USA; Office of the Clinical Director and Medical Genetics Branch, National Human Genome Research Institute, 10 Center Drive, Bethesda MD USA; Division of Clinical Research, National Institute of Neurological Disorders and Stroke, 6001 Executive Blvd, Rockville, MD, USA; Ophthalmic Genetics and Visual Function Branch, National Eye Institute, 10 Center Drive, Bethesda MD, USA; Ophthalmic Genetics and Visual Function Branch, National Eye Institute, 10 Center Drive, Bethesda MD, USA; Speech Language Pathology Section, Rehabilitation Medicine Department, Warren Grant Magnuson Clinical Research Center, 10 Center Drive Bethesda MD USA; Neurotology Branch, Division of Intramural Research, National Institute on Deafness and Other Communication Disorders, 10 Center Drive, Bethesda, MD USA; Inova Children’s Cardiology, 8260 Willow Oaks Corporate Drive; suite 400; Fairfax, VA, 22031; Nutrition Department, Warren Grant Magnuson Clinical Research Center, 10 Center Drive Bethesda MD USA; Office of the Clinical Director and Medical Genetics Branch, National Human Genome Research Institute, 10 Center Drive, Bethesda MD USA; Office of the Clinical Director and Medical Genetics Branch, National Human Genome Research Institute, 10 Center Drive, Bethesda MD USA; Office of the Clinical Director and Medical Genetics Branch, National Human Genome Research Institute, 10 Center Drive, Bethesda MD USA; Office of the Clinical Director and Medical Genetics Branch, National Human Genome Research Institute, 10 Center Drive, Bethesda MD USA; Office of the Clinical Director and Medical Genetics Branch, National Human Genome Research Institute, 10 Center Drive, Bethesda MD USA; Office of the Clinical Director and Medical Genetics Branch, National Human Genome Research Institute, 10 Center Drive, Bethesda MD USA; Office of the Clinical Director and Medical Genetics Branch, National Human Genome Research Institute, 10 Center Drive, Bethesda MD USA; Office of the Clinical Director and Medical Genetics Branch, National Human Genome Research Institute, 10 Center Drive, Bethesda MD USA; Sleep and Neurodevelopment Service, National Institute of Mental Health, 10 Center Drive, Bethesda MD USA; Neurotology Branch, Division of Intramural Research, National Institute on Deafness and Other Communication Disorders, 10 Center Drive, Bethesda, MD USA; Genetics and Metabolism, Children’s National Hospital, 111 Michigan Avenue NW, Washington DC USA; Ophthalmic Genetics and Visual Function Branch, National Eye Institute, 10 Center Drive, Bethesda MD, USA; Department of Radiology and Imaging Sciences, Warren Grant Magnuson Clinical Research Center, 10 Center Drive, Bethesda, MD, USA; Program in Neuroradiology, Children’s National Hospital, 111 Michigan Avenue NW, Washington DC USA; Radiology and Pediatrics, The George Washington University School of Medicine and Health Sciences, 2300 I St NW, Washington DC USA; Neurodevelopmental and Behavioral Phenotyping Service, National Institute of Mental Health, 10 Center Drive, Bethesda MD USA; Office of the Clinical Director and Medical Genetics Branch, National Human Genome Research Institute, 10 Center Drive, Bethesda MD USA

## Abstract

**Purpose.:**

GM1 gangliosidosis (GM1) is an ultra-rare lysosomal storage disease caused by pathogenic variants in galactosidase beta 1 (*GLB1*; NM_000404), primarily characterized by neurodegeneration, often in children. There are no approved treatments for GM1, but clinical trials using gene therapy (NCT03952637, NCT04713475) and small molecule substrate inhibitors (NCT04221451) are ongoing. Understanding the natural history of GM1 is essential for timely diagnosis, facilitating better supportive care, and contextualizing the results of therapeutic trials.

**Methods.:**

Forty-one individuals with type II GM1 (n=17 late infantile and n=24 juvenile onset) participated in a single-site prospective observational study. Here, we describe the results of extensive multisystem assessment batteries, including clinical labs, neuroimaging, physiological exams, and behavioral assessments.

**Results.:**

Classification of 37 distinct variants in this cohort was performed according to ACMG criteria and resulted in the upgrade of six and the submission of four new variants to pathogenic or likely pathogenic. In contrast to type I infantile, children with type II disease exhibited normal or near normal hearing and did not have cherry red maculae or significant hepatosplenomegaly. Some older children with juvenile onset developed thickened aortic and/or mitral valves with regurgitation. Serial MRIs demonstrated progressive brain atrophy that were more pronounced in those with late infantile onset. MR spectroscopy showed worsening elevation of myo-inositol and deficit of *N*-acetyl aspartate that were strongly correlated with scores on the Vineland Adaptive Behavior Scale and progress more rapidly in late infantile than juvenile onset disease.

**Conclusion.:**

The comprehensive serial phenotyping of type II GM1 patients expands the understanding of disease progression and clarifies some common misconceptions about type II patients. Findings from this 10-year endeavor are a pivotal step toward more timely diagnosis and better supportive care for patients. The wealth of data amassed through this effort will serve as a robust comparator for ongoing and future therapeutic trials.

## Introduction

GM1 gangliosidosis (GM1) is an ultra-rare lysosomal storage disease caused by pathogenic variants in galactosidase beta 1 (GLB1; NM_000404) that result in a deficiency of lysosomal beta-galactosidase.^1,2^ Without sufficient enzyme activity, GM1 ganglioside and related glycoconjugates build to toxic levels, particularly in the central nervous system.^3–5^ The disease is primarily characterized by neurodegeneration, but systemic involvement is also present and varies across disease types and among individual patients, resulting in considerable phenotypic complexity.

The phenotype of GM1 gangliosidosis exists as a spectrum of severities that are inversely related to the residual enzyme activity and the age of symptom onset. For convenience, the phenotypic spectrum has been divided into three types.^6,7^ Type I, the infantile form (MIM #230500) is the most severe and is characterized by a developmental plateau and regression by 4–6 months of age and rapid disease progression that includes severe hypotonia, spasticity, seizures, deafness, blindness and decerebrate rigidity.^8–10^ Infantile patients with multi-organ dysfunction, including hepatosplenomegaly, cardiomyopathy, skeletal dysplasia, coarse facial features, cherry red maculae and extensive Mongolian spots^11–16^, typically have a life expectancy of 2–3 years.^17,18^ Like other lysosomal storage diseases, type I GM1 can also present as nonimmune hydrops fetalis.^10,19^ Type II (MIM #230600) is less severe and has later onset than type I, and based on the timing of observable symptom onset can be further classified into late infantile (around 1 year of age) and juvenile onset (3 – 5 years).^6,20^ Type III GM1 gangliosidosis (MIM #230650), the adult form, is the least severe. Symptoms typically emerge in the second or third decade of life.^21,22^

The available descriptions of the type II phenotype, which have recently included a wider variety of genetic backgrounds, involve developmental stagnation that evolves into ataxia, dystonia, dysarthria, and skeletal changes.^23–25^ A small cases series^26^ noted dysphagia as a leading symptom, but lower than expected incidence of visceromegaly and no facial dysmorphisms or cherry red maculae. Progressive brain atrophy and/or white matter changes on magnetic resonance imaging and progressive deficits of *N*-acetylaspartate on magnetic resonance spectroscopy have been described in type II patients.^16,27^ Clinical laboratory studies describe borderline to mildly elevated aspartate aminotransferase with normal alanine aminotransferase levels.^25^ Low bone mineral density without fractures has been documented for both subtypes, and odontoid hypoplasia has been proposed as a hallmark in late infantile disease.^28^ The largest study to date, a retrospective cohort of 41 individuals^29^, largely supported the accumulated findings. However, the available data on the natural history of type II GM1 are from samples that are either small, retrospective, or cross-sectional, which limits our understanding of the progression of disease within individual and variability therein.

Thus, we sought to add to the existing literature by conducting prospective, repeated, and comprehensive assessments of 41 participants. The aims of this report are threefold: (1) to highlight the presenting signs and symptoms of type II disease in hopes of contributing to timely diagnosis, (2) to describe the course and variability of disease facilitating better supportive care, and (3) to chronical the natural progression of disease that can serve as a control cohort for therapeutic trials.

## Methods

### Participants

Individuals with a confirmed diagnosis of GM1 gangliosidosis type II were enrolled in National Institutes of Health (NIH) clinical protocol 02-HG-0107, “Investigation of Neurodegeneration in Glycosphingolipid Storage Disorders” (NCT00029965). This study was approved by the NIH Institutional Review Board, and informed consent or assent where appropriate was obtained from all participants. Diagnosis was confirmed by review of enzyme determinations and/or biallelic pathogenic variants in *GLB1* in a CLIA-certified laboratory. Medical history and physical examinations were used to classify participants as either late infantile or juvenile onset type II GM1 gangliosidosis. A subset of the participants in this study were represented in two previous reports from this research group.^28,30^

### Clinical Assessments

This study involved in-person visits conducted at the National Institutes of Health Clinical Center (Bethesda, MD), virtual assessments conducted via phone or videoconference, and parent questionnaires completed remotely. Following an initiation visit, participants were invited to return to the Clinical Center at intervals of 1–2 years. At each study visit, participants underwent comprehensive evaluations with a multidisciplinary team, including blood, urine and/or CSF collection, abdominal ultrasound, echocardiogram (ECHO), electrocardiogram, electromyography/nerve conduction velocities, consultation with a physiatrist, audiogram and auditory brainstem response (ABR) testing, ophthalmologic evaluation, electroencephalogram, magnetic resonance imaging of the brain, speech/language assessment including a swallow study when indicated, and neurodevelopmental testing with a psychologist. Additional details about methodology are provided in the Supplementary Methods. Participants continued routine clinical care at their home institutions, and medication compliance and its effect on disease was not measured. General medication information is summarized in Table 1 and in the Supplementary Materials. Participation in assessment was voluntary and not all patients underwent all evaluations at any given visit. Unless otherwise indicated, clinical laboratory tests were performed in the NIH Clinical Center Department of Laboratory Medicine, a CLIA-certified facility supporting the NIH Clinical Center.

### Analytic Approach

The goal of this study is to describe the course and variability of the signs and symptoms of type II GM1. Given the differential course of illness, in most cases results are reported separately for the late infantile and juvenile onset groups. The available longitudinal data are summarized graphically to illustrate the course of illness. However, the sample size could not support the appropriate inferential models (e.g., generalized linear models). For this reason, we focus on the descriptive analysis (frequencies for categorical data, median and interquartile ranges for ordinal or skewed data, and means and standard deviations for normally distributed data). To additionally support interpretation about the course of the disease using cross-sectional information, for quantitative outcomes we provide Spearman correlations with chronological age (ρ_age_), or for categorical outcomes the distribution of age within each level.

## Results

### Participant Characterization

**Figure 1.**
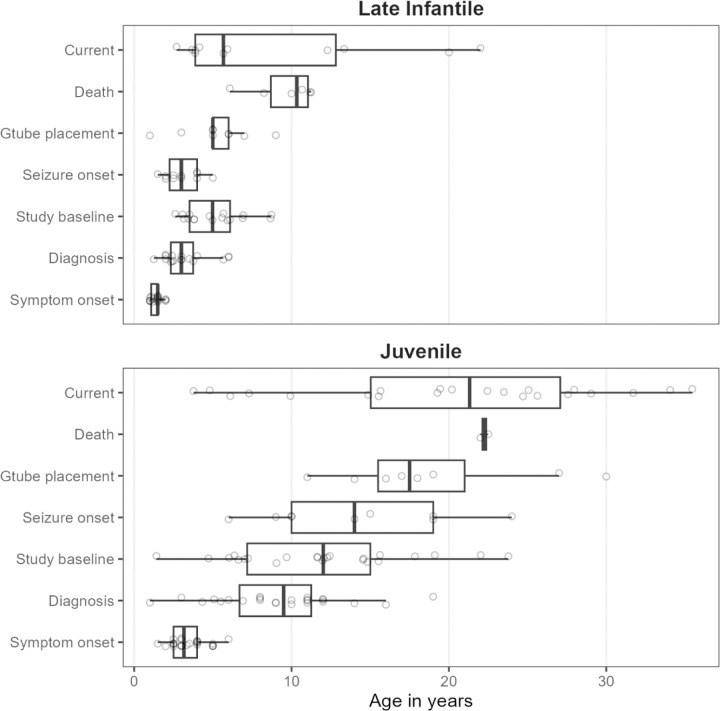
Summary of participant data. The timing of disease milestones and study participation are plotted. “Symptom onset” refers to the parent-reported age of first symptoms, which were either motor or speech-related. “Current age” refers to the age of last contact for participants who were alive. For most participants, this was August 2023, but for those who could not be recontacted it was the last visit date and for those enrolled in the gene therapy trial, it was the enrollment date of that study.

In total, 41 individuals with GM1 gangliosidosis type II were evaluated, including eight sibling pairs or trios (Table 1). Participants were classified as late infantile (10 female, 7 male) or juvenile (14 female, 10 male) onset. Figure 1 summarizes the timing of key events.

#### Genotype.

Likely causative variants were identified in *GLB1* in all individuals enrolled in the study and classified according to ACMG guidelines^31^ and modified criteria.^32–35^ Most participants were compound heterozygous for pathogenic (PATH) or likely pathogenic (LPATH) variants in GLB1 (late infantile: 12, 71%; juvenile: 22, 96% of 23 who were genotyped). In total, 37 variants were identified and classified; 30 were classified as PATH and seven as LPATH (see Supplementary Tables S1). In ClinVar, six variants were upgraded from VUS to LPATH, and four previously unreported variants were submitted, all classified as PATH or LPATH.

#### Symptom onset and diagnosis.

Retrospective report of developmental milestones was available for 16 (94%) late infantile and 24 (100%) juvenile onset participants. Those with late infantile onset usually exhibited delay or non-acquisition of major milestones (sitting, walking, first words), subsequently losing whatever skills had been acquired. However, the juvenile onset group usually attained these milestones on time relative to population norms and while loss was less common than for late infantile onset, it was not rare (Supplementary Figure S1). Most participants in both groups experienced both motor and speech-related initial presenting symptoms (Table 1). The median [IQR] time from symptom onset to diagnosis in probands (i.e., excluding siblings) was 1.53[0.96, 2.13] years for late infantile probands (n=12) and 5.5[3.42,8.75] years for the juvenile probands (n=18).

### Physical Metrics

#### Ophthalmology.

Seventeen (100%) of the late infantile participants had at least one ophthalmological evaluation; the most recent result for each participant is reviewed here. Among the late infantile cohort, only six were able to complete formal acuity testing with a Snellen equivalent, with an average LogMAR acuity of 1.20 (equivalent to 20/314) (the remaining were recorded as no or questionable blink to light, n=3; blink to light, n=4; occasional fix/follow, n=1; fix/follow, n=1; and central/steady/maintained or central/steady/unmaintained, n=2). Ptosis was rare and mild-to-moderate (n=1, 6%), but strabismus and nystagmus were common (n=17, 100% and n=9, 53%, respectively). Mild (n=8, 47%) and moderate (n=1, 6%) corneal clouding (hazing) was observed. On retinal exam no patients had a cherry-red maculae, but optic nerve pallor/atrophy was noted in four (24%) late infantile patients. The mean spherical equivalent of the refractive error was +2.62±2.53 Diopters (n=17). Cortical/neurological vision impairment was notable in 12 (71%).

Twenty-one (88%) juvenile participants had at least one ophthalmological evaluation. Their visual acuity ranged between 20/20 and 20/200; the average LogMAR acuity for the better seeing eye was 0.34 (equivalent to 20/44) (n=19 with available data). No nystagmus was noted in the juvenile cohort, but mild-to-moderate ptosis (n=4, 19%) and strabismus (n=5, 24%) were observed. Corneal clouding/hazing (mild, n=9; moderate, n=3) was common. Retinal exam was generally normal, with occasional granular retinal periphery which may be normal variant, and optic nerves were mostly normal (only the oldest patient exhibited mild degrees of pallor). No cherry red maculae were observed. Refractive error was mostly within normal range for age (mean spherical equivalent of the refractive error, +1.10 ± 2.64 Diopters). Finally, no cortical/neurological visual impairment was observed.

#### Audiology.

All participants received at least one audiological evaluation and their most recent and most complete assessment was used for cross-sectional summary (Supplementary Table S2). Peripheral hearing sensitivity was within normal limits for most participants (late infantile: n=15, 88%; juvenile: n=22, 92%), and the available longitudinal data (late infantile: n=6, 35%; juvenile: n=16, 67%) indicated that it was stable. There was no evidence of gross middle ear dysfunction in any participant. Among those who received neurodiagnostic ABR testing, the results were within normal limits for eight of 15 (53%) late infantile and 14 of 22 (64%) juvenile participants. Abnormalities included small V/I amplitude, poor neural synchrony, and/or retrocochlear auditory dysfunction. No gross changes were noted in the available longitudinal data, indicating stability.

#### Video fluoroscopic assessments of swallowing (VFSS).

For 14 (82%) of the late infantile group and 23 (96%) of the juvenile group, VFSS was prompted by clinical interview and assessment (age in years at assessment median[IQR]: late infantile, 5 [3.5, 5.9]; juvenile, 11.8 [7.6, 14.7]). In the juvenile group, VFSS scores (see Supplementary Table S6) indicated little risk of aspiration (median[IQR]: 5 [4, 5], ρ_age_ =−0.28) and safe and efficient swallowing (median[IQR]: 4 [3.5, 4.5], ρ_age_ =−0.53). Dietary restrictions were generally absent or minimal (median[IQR]: 4 [4, 5], ρ_age_ =−0.68), and only three participants (aged 15 – 24 years) had maximal or moderate restriction. Aspiration (median[IQR]: 4 [0.25, 5], ρ_age_ =−0.35) and swallowing (median[IQR]: 3 [0.25, 4.75], ρ_age_ =−0.36) scores were slightly more severe and variable in the late infantile group. For the majority of late infantile participants (n=9 of 14), dietary restrictions were minimal or none, but the remaining five participants had maximal restriction (median[IQR]: 4 [2, 5], ρage=−0.44). The negative correlations with age reflected more severe ratings in older participants than in younger participants, but the limited within-participant longitudinal data indicated stability over the observation period.

#### Cardiology.

**Figure 2.**
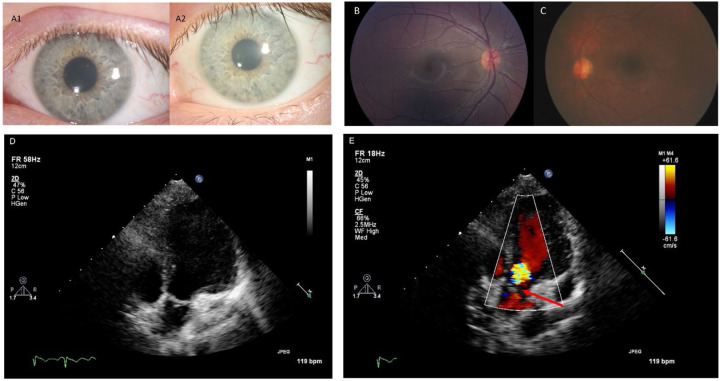
Selected ophthalmology and cardiology results. Panels A1 and A2: Corneal slit-lamp photographs from the same Juvenile GM1 patient showing slow progression of corneal clouding over a five-year period. Panel B: Left eye Topcon color fundus photograph from a Juvenile GM1 patient showing an essentially normal fundus with no evidence of macular or optic nerve head pathology. Panel C: Right eye RetCam fundus photography of a late-infantile GM1 patient showing normal macula and optic nerve with minimal retinal vascular tortuosity. Panels D and E: Two dimensional and color transthoracic echocardiographic imaging of the left ventricular outflow tract from a single Juvenile GM1 patient. The red arrow points to aortic valve leaflet thickening with associated aortic regurgitation. These images are representative of the abnormal thickening of the aortic valve leaflets with associated aortic regurgitation observed in three unrelated Juvenile GM1 patients (23% of those who received ECHO; an additional five participants had aortic and/or mitral valve leaflets that were qualitatively thickened for age but without regurgitation or stenosis).

ECHO was performed for nine (53%) late infantile participants, revealing only one with mitral valve prolapse and associated mitral regurgitation. No evidence of aortic valve leaflet thickening or dilated or hypertrophic cardiomyopathy was observed. One participant did have borderline aortic root dilation. Among 13 juvenile participants receiving ECHO, three (23%) had aortic valve leaflet thickening with associated aortic valve regurgitation ranging from trace to moderate in severity (Figure 2). One of these affected individuals also had associated trivial aortic stenosis. Five additional participants had aortic and/or mitral valve leaflets that were qualitatively thickened for age without any associated valvular regurgitation or stenosis but did not reach a threshold to be commented upon by the clinical cardiology teams. Biventricular function and chamber dimensions remained within acceptable limits for age in all cases. No clinically significant EKG abnormalities were observed among the participants who were evaluated (late infantile: n=9, 53%; juvenile: n=15, 63%).

#### Abdominal Ultrasound.

At least one abdominal ultrasound was performed in nine late infantile (53%; age median[IQR] = 3.5[3.8, 6.1]) and 17 juvenile participants (71%; age median[IQR] = 7.2[12.3, 19.9]). The majority of assessments were normal; at the first available timepoint, mild enlargement was observed for two (22%) late infantile participants and two (13%) juvenile participants. All four participants with radiologically abnormal findings had abnormal AST levels, one had abnormal ALT, and none had abnormal GGT. The two late infantile participants with enlargement had no follow-up, but each of the juvenile participants had a 1-year follow-up showing hepatomegaly for one and a return to normal for the other. None of the participants with hepatomegaly had splenomegaly, however, minimal-to-mild splenomegaly was observed at the first assessment for two other late infantile participants (22%) and one other juvenile participant (6%). No follow-up imaging was available for these three individuals. An additional juvenile participant had developed mild splenomegaly 1 year after the first evaluation.

#### Mobility.

At least one mobility assessment was available for all participants (Supplementary Figure S2). In the late infantile group, baseline floor mobility scores were variable, with a median score of “sits without support” (median[IQR] = 3 [0, 5]), and older participants had worse scores than younger participants (ρ_age_ = −.47). In the juvenile group, baseline upright mobility scores were less severe, with a median score of “independent ambulation, may be unsteady” (median[IQR] = 4 [1.75, 4]). Scores in the juvenile group were also strongly correlated with age (ρ_age_ = −.74). The longitudinal data indicated worsening over time within person for both onset groups (Supplementary Figure S2). To support the validity of the mobility assessment, we compared the scores with the Vineland-3 Gross Motor GSVs for 21 participants with contemporaneous evaluations, finding strong positive Spearman correlations (floor, ρ=0.87; upright, ρ=0.75).

### Laboratory Studies

The β-gal enzyme activity in available serum for late infantile (n=11, 65%) and juvenile (n=21, 88%) patients ranged from 0–5% (median[IQR] = 0.028[0.014, 0.04]) of the pediatric control sample (Supplementary Figure S3). Enzyme activity in available CSF for late infantile (n=5, 29%) and juvenile (n=13, 54%) patients ranged from 2–8% (median[IQR] = 0.05[0.026, 0.07]) of the same pediatric controls. Serum and CSF were not meaningfully correlated with one another, though this was likely due to the extremely limited ranges (Supplementary Figure S3).

Blood samples from all participants were evaluated for complete blood count with differential, complete metabolic panel (hepatic panel, creatinine kinase, lipid panel), iron panel, thyroid panel, vitamin D, prothrombin time/partial thromboplastin time, and lactate dehydrogenase. Within the cross-sectional data (first available visit), all parameters were in the normal pediatric ranges except for several liver enzymes: aspartate aminotransferase (AST) was elevated for 13 (76%) late infantile and seven (29%) juvenile onset patients, alanine aminotransferase (ALT) was elevated for three (18%) late infantile two (8%) juvenile onset patients, and gamma-glutamyl transferase (GGT) was elevated for six (35%) late infantile and six (25%) juvenile onset patients (Supplementary Table S3). The longitudinal data suggested stability in these parameters, indicating that these parameters did not change systematically within individuals over the observation period (Supplementary Figure S4).

### Electrodiagnostic and Neuroimaging

#### Electroencephalography (EEG).

Twelve (71%) late infantile participants received at least one outpatient ambulatory EEG; the first available EEG is summarized here (age at first assessment, median 5.3[3.4, 6.9] years). Most (n=10, 83%) of these EEGs were abnormal: background abnormalities (including low overall voltage, slow posterior dominant rhythm or abnormal background slowing) were noted in nine (90%), focal or persistently rhythmical brief slowing was noted in four (40%) (delta-range predominant in two, theta-range predominant in two), and four (40%) had epileptiform activity (typically sharp waves over temporal and sometimes frontal head regions). One of the participants with a normal EEG at the first visit had possible progression with the lack of a normal posterior basic rhythm in an overnight EEG 1 year later; the other participant did not have any follow-up EEGs. Four of the participants with an abnormal first EEG had repeated ambulatory or overnight assessments, with consistently abnormal results.

The 18 (75%) juvenile participants with at least one EEG had a median age of 15.2[11.7, 17.9] years at the earliest available assessment. All 12 (67%) abnormal EEGs had background abnormalities, five (42%) had focal or persistently rhythmical bursts of brief slowing (theta-range in four patients, and alpha-range in one patient), and one (6%) had epileptiform activity (typically sharp waves over temporal and sometimes frontal head regions). Among the six participants with normal EEGs at the earliest assessment, two had an abnormal EEG (background abnormalities, similar to those noted in the late infantile onset group) at a later timepoint, one had two more normal EEGs, and three had no other EEGs. Among the 12 participants with abnormal EEGs at the earliest assessment, six had no follow-up ambulatory or overnight EEG, four had two or more follow-up ambulatory or overnight EEGs that were consistently abnormal, and two had both normal and abnormal follow-up EEGs.

#### Structural magnetic resonance imaging (MRI) of the brain.

**Figure 3.**
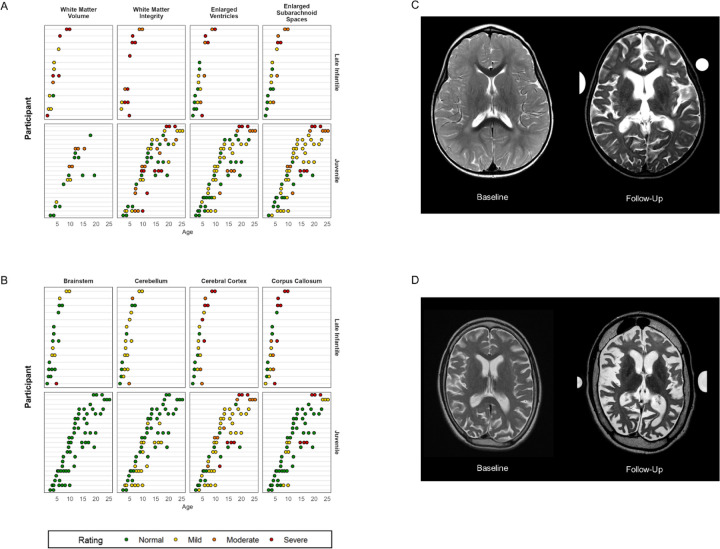
MRI qualitative ratings for all participants. Panels A and B: Qualitative MRI ratings are shown per MRI per person (Y-axis) by age in years (X-axis). Late infantile onset data are shown in the top rows and juvenile onset data in the bottom row. Cerebral white matter could only be assessed in n=8 participants with late infantile onset, as the others lacked the pulse sequences necessary for proper evaluation. Panel C: Serial MRIs from a single patient with late infantile onset at baseline and a follow-up 2.5 years later (ages redacted per MedArXiv requirements). Panel D: Serial MRs from a single patient with juvenile onset baseline and a follow-up 8 years later (ages redacted per MedArXiv requirements).

Out of 14 (82%) of the late infantile cases with at least one MRI, 11 (79%) presented with or developed atrophy of the cerebellum. Two of these cases were described as moderate, and nine as mild atrophy. Eleven (79%) late infantile participants presented with or developed cerebral cortical atrophy, including four severe and three moderate. A mid sagittal T1-weighted image was not available for one participant, so the size of the brainstem and corpus callosum could not be evaluated in this case. Of the remaining 13 cases, five (38%) presented with or developed brainstem atrophy, including one severe, and nine (69%) with atrophy of the corpus callosum, including five severe and two moderate. Cerebral white matter could be assessed in only eight cases (other cases lacked the pulse sequences necessary for proper evaluation of myelination) all showed abnormalities of myelination, half (n=4) of which were severe. Using the latest available observation for each person, the qualitative atrophy scores in each of these regions was correlated with age, such that older participants had worse ratings (cerebral cortex, ρ_age_ = 0.79; corpus callosum, ρ_age_ = 0.72; see also Figure 3). Longitudinal data suggested that most participants worsened over time (Figure 3).

Twenty-one (88%) of the juvenile participants had at least one MRI. Six (29%) presented with or developed mild atrophy of the cerebellum; 17 (81%) with cerebral cortical atrophy, including three severe and three moderate; and five (24%) with atrophy of the corpus callosum, including two severe. Out of 19 cases for which white matter could be evaluated, 13 (68%) presented with or developed white matter injury, including four severe and five moderate. The brainstem appeared normal in all cases. No consistent cross-sectional trends between age and atrophy were observed using the most recent MRI, and the available longitudinal data indicated general stability for most participants during the observation period (Figure 3).

#### Magnetic resonance spectroscopy (MRS) of the brain.

**Figure 4.**
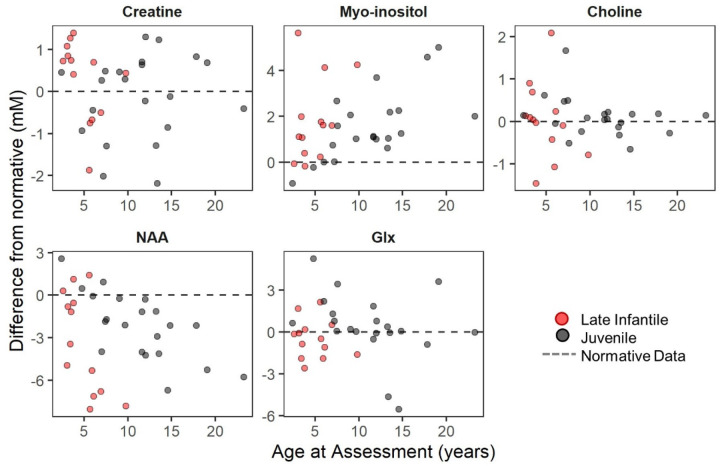
Age progression of metabolite concentration (mM) in LCSO relative to normative expectations. NAA: *N*-acetyl aspartic acid. Glx: Glu+Gln+GABA. Red = late infantile onset; black = juvenile onset. Earliest available observation per person (Late Infantile, n = 13; Juvenile, n = 21) is plotted. Points show difference in concentration from age expectation, which is illustrated by the dotted line at zero.

At least one visit with MRS was available for 13 (76%; n=2 with two visits) of the late infantile group and 21 (88%; n=14 with two, three, or four visits) of the juvenile group. For both groups, the average concentration of creatine, choline, and glutamine+glutamate+gamma-aminobutyric acid (glx) measured in the left centrum semi-ovale were similar to age-normative expectations, but both myo-inositol (elevated) and *N*-acetylaspartate+*N*-acetylaspartyl glutamate (NAA; decreased) were abnormal (**Figure 4 and** Supplementary Table S4). Within the cross-sectional data, older age was associated with increased deviation from normative values for both myo-inositol (late infantile ρ=0.28; juvenile ρ=0.68) and NAA (late infantile ρ=−0.59; juvenile ρ=−0.74) (see Supplementary Table S4a). The juvenile cohort had sufficient data to attempt formal longitudinal modeling (see Supplementary Methods) (Supplementary Figure S5). Consistent with the cross-sectional analysis, older participants tended to have greater excess of myo-inositol (t=2.63, p=0.016) and greater deficit of NAA (t=−3.56, p=0.002) than younger participants (see Supplementary Table S4b). However, a trend towards detectable within-person change relative to normative expectations during the observation period was observed only for myo-inositol (t=2.01, p=0.068) and not for NAA or other metabolites.

### Neurodevelopmental Assessments

#### Speech evaluation.

All participants in both groups participated in at least one speech evaluation (see Supplementary Table S6); the earliest available assessment is reported here (median[IQR] for age at assessment: late infantile, 5 [3.5, 5.9]; juvenile, 11.8 [7.6, 14.7]). Most (n=13, 76%) of the late infantile onset group had the most severe score of zero for both speech and language ratings (median[IQR] scores: speech, 0 [0, 0]; language, 0 [0, 0]). Three late infantile participants had longitudinal data, and their scores were at the floor for all visits. The juvenile onset group had a severe speech presentation (median[IQR]= 2 [1, 3]) and was deficient in most areas of language (median[IQR]= 1 [1, 1]); both of these scores were more severe in older participants than in younger participants (correlation with age: Speech ρ = −0.60; Language ρ = −0.53). The limited juvenile within-subject longitudinal data indicated stability (Supplementary Figure S6).

#### Adaptive behavior.

**Figure 5.**
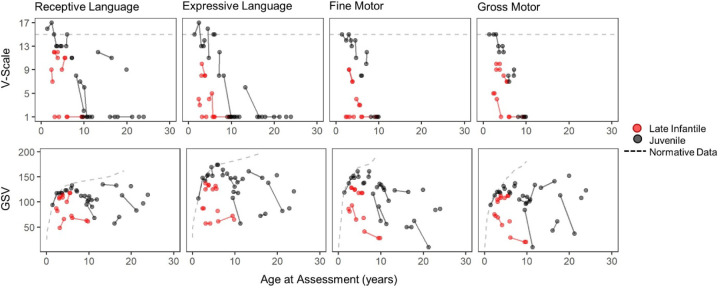
Selected Vineland-3 subdomain scores, expressed as V-scale (top row) and growth scale value (GSV; bottom row). Red = late infantile onset (n = 8); black = juvenile onset (n = 14). Solid lines connect observations from the same person. V-scale scores have a population mean of 15 (dotted line) and SD of 3. The population-level distribution of GSV is not defined, but the median value per age equivalent in the normative sample is plotted (dotted line). Gross and Fine Motor subdomain V-scale scores are available only through age 9 years.

Most late infantile (n=14, 82%) and juvenile (n=23, 96%) participants had at least one administration of the Vineland Adaptive Behavior Scales (see Supplementary Figure S7 for longitudinal data). The cross-sectional dataset was created using the participant’s first Vineland-3 (Late infantile, n=8; Juvenile, n=14), or their first Vineland-II (Late infantile, n=6; Juvenile, n=9) if the third edition was not given. Most participants in both groups (Late infantile: n=12 of 14; Juvenile: n=19 of 23) had Adaptive Behavior Composite (ABC) standard scores below the 2.5th percentile. The median ABC scores were in the moderately impaired range for both groups, and floor effects were common for the juvenile onset group, but not the late Infantile group (**Table 2; see also** Supplementary Table S5). Strong negative correlations were observed between ABC scores and chronological age in both groups. Figure 5 illustrates that floor effects in V-scale scores dramatically obscured the observed between- and within-participant variability in performance, and that the growth scale values (GSVs) were sensitive to that variability, making the latter more useful as outcomes in future trials.

**Figure 6.**
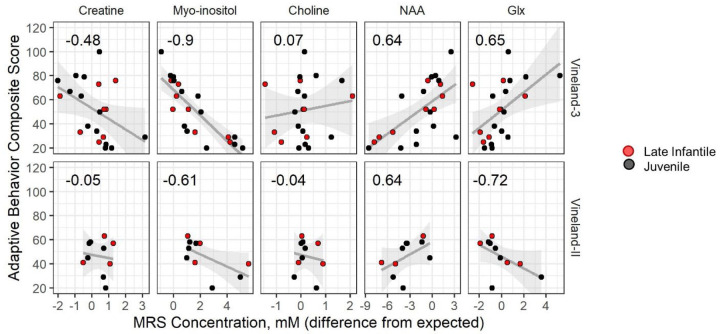
Correlation between LCSO MRS concentration (mM, deviation from expected) and Vineland scores. Cross-sectional data are plotted; the earliest available Vineland-3 was selected for each participant (top row) and if the participant had no Vineland-3 then the earliest available Vineland-II was used (bottom row). Each panel illustrates the Spearman correlation (value inset) for the relationship between Vineland Adaptive Behavior Composite (ABC) and the MRS concentrations (mM) in LCSO, each expressed as difference from age expectations (columns). Correlations are shown separately for the second and third editions of the Vineland as the scores are not combinable. Points are colored by onset group, black is late infantile and red is juvenile. Gray lines and shaded area represent the linear regression line and 95% confidence interval.

We explored the relationship between MRS, a potential severity biomarker, and adaptive behavior scores. Given that the MRS concentrations are expressed as deviance from age-expected values, we compared those to the age-norm-referenced ABC standard scores. Using the cross-sectional data (first available Vineland-3, n=8 late infantile and n=13 juvenile, otherwise first available Vineland-II, n=4 late infantile and n=6 juvenile) we calculated Spearman correlations between ABC and MRS (Figure 6). Myo-inositol elevations (ρ_VABS3_=−0.90, ρ_VABSII_=−0.61) and NAA deficits (ρ_VABS3_=0.64, ρ_VABSII_=0.64) were strongly correlated with more impaired adaptive behavior.

## DISCUSSION

The comprehensive serial phenotyping of type II GM1 patients in this 10-year prospective study expands our understanding of the disease spectrum. While the classification of disease into late infantile and juvenile onset parses some heterogeneity in presentation and progression, considerable variability remained even within the subtypes. Although small sample size precludes a genotype-phenotype investigation, it seems reasonable to assume that this heterogeneity is related to the high degree of genetic variability and compound heterozygosity among the 165 pathogenic and 112 likely pathogenic variants in *GLB1* described in this disease.^36^ Using ACMG criteria we classified each of the 37 variants in our cohort; all of these were classified as pathogenic or likely pathogenic. The classification is based on a deeply phenotyped cohort and low enzyme activity in serum and CSF compared to an age-matched pediatric control group. We anticipate that this classification will aid clinical laboratories in variant interpretation when the phenotype or enzyme testing results are unavailable.

As is often documented for other ultra rare genetic conditions associated with neurodevelopmental disability^37^, delay, absence, or loss of major milestones such as walking and talking were typically the first observable symptoms of type II disease. These symptoms are perhaps more subtle, and certainly less specific, than the various physical manifestations described as cardinal symptoms of type I disease.^11–16^ This may explain the long delay between symptom onset and diagnosis in this study – 1.5 years for late infantile onset and 5.5 years for juvenile onset probands. Loss of major milestones, which occurs very rarely among healthy children, was common in both late infantile and juvenile onset groups. Further, the parent-reported and direct assessments in this study converged upon a clinical picture of ongoing deterioration in whatever gross motor and language abilities had been attained for both onset groups, though the progression was more rapid among late infantile patients. Thus, loss or deterioration in motor or speech domains is a particularly salient indicator for genetic testing, even if milestone attainment had previously been normal.

The combination of retrospective reports and prospective evaluations in this study yielded valuable insights into two domains of clinical concern in GM1, feeding and seizures. Consistent with other reports^29^, gastronomy tubes were common among participants with late infantile onset, most of which were placed in early childhood. Only about 1/3 of juvenile onset participants received a gastronomy tube, but the placement age was usually late in the second decade of life. The prospective formal assessment of swallowing behaviors revealed that older participants tended to have worse swallowing abilities. Because feeding is a clinically salient symptom domain, these findings support the use of gastronomy tube placement and/or more precise quantification with swallowing evaluations as outcome measures in future study.

Seizures are a hallmark of GM1, and it is known that most patients will develop clinical or electrographic seizures over the course of their disease. Indeed, nearly all late infantile onset participants had a history of seizures prior to enrollment, with several experiencing seizures prior to the GM1 diagnosis, and all participants who developed seizures did so by age 5 years. Seizures were less common, but not rare, for the juvenile onset group. However, the onset age was extremely variable, ranging from 6 to 24 years. While the retrospective report of clinical diagnosis of seizure is important, it remains possible that atypical presentation may prevent the timely diagnosis of seizures in individuals with GM1. Because the deep phenotyping of this study included prospective EEG evaluation, we were able to document that 83% of late infantile and 67% of juvenile EEGs were abnormal either due to background abnormalities, focal slowing, or epileptiform activity.

The prospective design of this study included evaluations of other areas thought to be impacted by GM1. Upon systematic ophthalmologic examination, no participants in this cohort exhibited cherry red maculae, though the extent of other ophthalmologic abnormalities appeared to be related to the severity of disease. Similarly, while hearing impairment has been described as a feature of Type I GM1^2^, the majority of Type II participants in this study had normal hearing sensitivity that was stable over time, with no middle ear dysfunction and normal ABRs. Thus, the absence of cherry red maculae or hearing impairment should not distract the diagnostic odyssey from the GM1 diagnosis.

Although cardiomyopathy is common in other LSDs such as Fabry disease and Pompe disease^38,39^ the results of the current study suggest that it is not a characteristic feature of type II GM1 gangliosidosis. About half of this cohort underwent cardiac ultrasound exam, yielding essentially normal results. However, three of the oldest juvenile onset patients had aortic valve leaflet thickening with regurgitation suggesting that for older type II juvenile onset patients, periodic assessment of cardiac function may be indicated.

Hepatosplenomegaly is a hallmark of some LSDs and in a recent retrospective natural history report was identified in the majority of early onset GM1 patients.^11,18,29^ Interestingly, it was not a major feature in our type II GM1 patients, as only mild or borderline enlargement observed in only a minority of patients upon systematic direct assessment. Still, liver enzymes GGT, ALT, and AST indicated some (stable) elevations in many patients. AST was elevated in all the participants with liver enlargement, but it was also elevated in most other late infantile onset patients and about 30% of the juvenile patients. Elevations in AST most often reflect liver dysfunction particularly in association with other liver enzymes ALT and GGT. However, AST evaluations can also reflect other sources including cardiac, muscular, hematologic, renal, and endocrine systems ^40,41^ and acute encephalopathy and seizures.^42^ The elevation of additional liver enzymes such as elevated GGT in 35% of the late infantile and 25% of the juvenile onset patients is likely a more accurate indicator of possible liver dysfunction. This would be an important consideration for AAV9 gene therapy for GM1 patients, as patients in other rare disease cohorts treated with systemic AAV have experienced liver toxicity.^43^

Of course, the brain is anatomically, chemically, and functionally the focal point of pathology in patients with GM1. A previous study^27^ (including some of the current participants) indicated that quantitatively defined atrophy was more severe among participants with late infantile onset compared to juvenile onset. In this larger cohort, qualitative ratings indicated myelin disruptions or abnormalities in all late infantile and most juvenile onset patients, in addition to considerable atrophy. From the perspective of clinical trial readiness, it seems plausible that some intervention may slow atrophy, but a reversal seems unlikely. Other indicators of neuronal status may therefore be useful as biomarkers of disease severity and/or response to treatment.

As suggested by others^27^, MRS may be fit for this purpose. In the current study, the quantification by MRS of six metabolites in the left centrum semiovale documented significant excess of myo-inositol (a measure of gliosis) and deficits in NAA (a measure of neuronal health). These findings are consistent with the neuronal loss and increased gliosis observed in murine and feline models of GM1 disease.^44–47^ A strength of this study was our ability to compare these putative biomarkers with both age and behavior, finding that abnormalities in both myo-inositol and NAA were related to age and adaptive functioning. Future work is needed to elucidate their viability as biomarkers for type II GM1.

In summary, although variability in type II GM1 gangliosidosis disease progression is evident in both subtypes we show here that no child escapes a progressive downward trajectory. This work builds upon the existing literature by using direct multisystem assessments over a long period of time within person, and a wide age range across individuals. Given the variability in type II GM1 gangliosidosis it will be important to design outcome measures for therapeutic trials that are sensitive to stability or subtle improvement, realizing that some features (e.g., cerebral atrophy) will not be reversible. Early diagnosis and newborn screening are therefore critical in identifying pre-symptomatic patients who have the greatest chance of deriving benefit from mechanism-modifying therapy. Fortunately, a collaboration of academicians, industry, and patient advocacy groups is actively engaged in this endeavor.^48^

## Supplementary Material

Supplement 1

Supplement 2

## Figures and Tables

**Table 1.** Participant characteristics. One participant in the Juvenile group was asymptomatic at enrollment, so age at diagnosis was less than age of symptom onset. The medication information is for the duration of the study only and does not distinguish the duration or dose (see Figure 1).

		Late Infantile Onset(n=17)	Juvenile Onset (n=24)	Full Sample (N = 41)
Female, n (%)		10 (59)	14 (58)	24 (59)
Race, n (%)	Asian	1 (6)	2 (8)	3 (7)
	Black/African American	1 (6)	1 (4)	2 (5)
	Multiracial	1 (6)	1 (4)	2 (5)
	Unknown	0	4 (17)	4 (10)
	White	14 (82)	16 (67)	30 (73)
Ethnicity, n (%)	Hispanic or Latino	0	5 (21)	5 (12)
	Not Hispanic or Latino	17 (100)	18 (75)	35 (85)
	Unknown	0	1 (4)	1 (2)
Country of residence, n (%)	Germany	0	1 (4)	1 (2)
	Poland	0	1 (4)	1 (2)
	United States	13 (76)	22 (92)	35 (85)
	Russia	1 (6)	0	1 (2)
	Sweden	3 (18)	0	3 (7)
Proband, n (%)		12 (71)	18 (75)	30 (73)
Age at consent (years)	Median [IQR]	4.98 [3.52, 6.09]	12.02 [7.18, 15.01]	7.03 [4.98, 12.27]
	Range	2.61 – 8.72	1.41 – 23.78	1.41 – 23.78
Age at symptom onset (years)	Median [IQR]	1.5 [1.08, 1.5]	3 [2.5, 4]	2.5 [1.5, 3.5]
	Range	1 – 2	1.5 – 6	1 – 6
Parent-reported symptoms at onset^a^, n (%)	Motor	15 (88)	16 (67)	31 (76)
	Speech	14 (82)	21 (88)	35 (85)
Age at diagnosis (years)	Median [IQR]	3 [2.33, 3.75]	9.5 [6.69, 11.25]	6 [3, 10]
	Range	1.25 – 6	1 – 19	1 – 19
Gastronomy tube, n (%)		9 (53%)	8 (33%)	17 (41%)
Age at G-tube, (years)		5 [5, 6]	17.5 [15.5, 21]	9 [5, 17]
Any miglustat during study, n (%)		4 (24)	10 (42)	14 (34)
Any tanganil during study, n (%)		2 (12)	2 (8)	4 (10)
Any seizure meds during study, n (%)		11 (65)	7 (29)	18 (44)

a Two juvenile participants are missing this information, so juvenile N = 22. Categories are not mutually exclusive.

**Table 2.** Vineland Adaptive Behavior Standard Scores at the First Visit. The first Vineland-II and/or first Vineland-3 assessment available for each person is summarized in this table. The Spearman correlation between the standard score and age is shown in the *ρ*_*age*_ column. Sample sizes and ages were: Late Infantile/Vineland-3: n = 8 (median age: 3.97 [3.06, 5.98]), Late Infantile/Vineland-II: n = 6 (median age: 4.09 [3.46, 4.91]); Juvenile/Vineland-3: n = 14 (median age: 9.59 [7.37, 17.39]), Juvenile/Vineland-II: n = 9 (median age: 17.29 [11.99, 19.38]). Motor excluded as standard scores are not available for all participants. Standard scores have a population mean of 100 and SD of 15 (floor = 20). IQR = interquartile range (25th – 75th percentile).

Group	Domain	Vineland-3				Vineland-II			

		Standard Score, median [IQR]	% ≥2SD below average	% at floor	ρ_age_	Standard Score, median [IQR]	% ≥2SD below average	% at floor	ρ_age_
Late Infantile	Composite	56.5 [32, 65.25]	75	0	−0.86	43 [39.25, 54.25]	100	0	−0.60
	Communication	43 [20, 65]	75	38	−0.85	43 [42, 51.5]	100	0	−0.49
	Daily Living	56.5 [38, 68.5]	75	0	−0.73	46 [43.75, 59.5]	100	0	−0.61
	Socialization	60 [35.25, 69.5]	75	0	−0.80	56 [48.25, 66.75]	100	0	−0.14
Juvenile	Composite	44 [24.5, 70]	71	21	−0.88	29 [26, 53]	100	22	−0.55
	Communication	36 [20, 68.5]	79	43	−0.80	28 [28, 52]	100	0	−0.77
	Daily Living	45.5 [20, 65.75]	79	36	−0.93	28 [25, 51]	100	0	−0.56
	Socialization	57 [30.25, 76.75]	57	21	−0.84	42 [37, 55]	100	11	−0.52

## Data Availability

The data described in this manuscript are available from the corresponding author upon reasonable request.
