# Supplementary material for: GM1 Gangliosidosis Type II: Results of a 10-Year Prospective Study
Source: medRxiv. 2024 Jan 4:2024.01.04.24300778. Preprint. [Version 1] doi: 10.1101/2024.01.04.24300778 (PMC10836125; doi:10.1101/2024.01.04.24300778)

medRxiv preprint doi: https://doi.org/10.1101/2024.01.04.24300778; this version posted January 4, 2024. The copyright holder for this preprint (which was not certified by peer review) is the author/funder, who has granted medRxiv a license to display the preprint in perpetuity.
This article is a US Government work. It is not subject to copyright under 17 USC 105 and is also made available for use under a CC0 license.

Manuscript Title: GM1 Gangliosidosis Type II: Results of a 10-Year Prospective Study

SUPPLEMENTARY METHODS

Symptom onset and milestones. Parents responded to a study-specific questionnaire asking about the nature and timing of milestone attainment (i.e., sitting, crawling, walking, talking) and the nature and timing of the earliest symptoms. Data from this questionnaire were combined with semi-structured clinical interview data from the neurodevelopmental assessment (see below). Finally, we attempted recontact via phone with participants in August 2023 to update the onset of symptoms (e.g., gastronomy tube placement) and acquisition or loss of milestones. Recontact data from participants who had enrolled in NCT03952637 were not included, as this information reflected disease state after the administration of gene therapy.

Ophthalmology. Eye exams were performed by pediatric ophthalmologists with ophthalmic genetics expertise. The ophthalmic assessment included a measurement of visual acuity with a Snellen chart or equivalent. When patient age, cognitive level, or cooperation precluded Snellen testing, Teller acuity was attempted to try and quantify visual acuity. The assessments of visual function are hierarchical, such that no blink to light reflects poor vision, followed by blink to light, occasional fix/follow, fix/follow, and central/steady/maintained or unmaintained. Achievement of the highest category indicates achievement of lower categories. Exams also included documentation of eyelid ptosis, ocular alignment, presence or absence of nystagmus, corneal and lenticular clarity, and fundus appearance including optic nerve head status. Where possible, clinical findings were supplemented with eye exams performed while the participant was under anesthesia for study related imaging procedures.

Audiology. Audiological testing included age- and ability- appropriate behavioral assessments of hearing for pure-tone and speech stimuli, measures of middle ear function (tympanometry), and noninvasive physiologic testing (distortion product otoacoustic emissions)

medRxiv preprint doi: https://doi.org/10.1101/2024.01.04.24300778; this version posted January 4, 2024. The copyright holder for this preprint (which was not certified by peer review) is the author/funder, who has granted medRxiv a license to display the preprint in perpetuity. This article is a US Government work. It is not subject to copyright under 17 USC 105 and is also made available for use under a CC0 license.

to assess cochlear health and supplement data from the behavioral assessment. For some participants, a neurodiagnostic auditory brainstem response (ABR) was performed; rarefaction and condensation click stimuli were presented separately at a high level (e.g., 85 dB nHL) with a low repetition rate (8.3 clicks/s). ABRs were interpreted using wave presence and morphology, absolute and interpeak latencies, and the wave V/I amplitude ratio.[1] However, due to poor patient cooperation, complete behavioral audiogram assessments were available for only 11 (46%) juvenile participants and no late infantile participants.

Video fluoroscopic assessments of swallowing (VFSS). When clinically indicated from historical data review (e.g., historical and current indicators of possible dysphagia, including weight loss, recent bronchitis, aspiration pneumonia, complaints of swallowing difficulties with one or more food textures, coughing while drinking, throat clearing after drinking, and/or complaints of food becoming stuck within the throat), video fluoroscopic assessments of swallowing (VFSS) were conducted systematically, assessing liquid, puree, and solid textures. Swallowing function was interpreted for appropriate safe swallowing. Following each study, the speech language pathologist summarized physiologic findings and applied a modified version of the American Speech-Language-Hearing Association / National Outcomes Measurement System (ASHA/NOMS) Dysphagia Scale, Diet Restrictions, and Modification Ratings[2] to rate the overall ability to swallow safely from 0 (most severe) to 5 (least severe). The extent of aspiration/laryngeal penetration was rated using a 0 (most severe) to 5 (least severe) ordinal scale adapted from the Rosenbek Penetration/Aspiration Scale[3] (PAS). These scales are described in Supplementary Table S8.

Abdominal ultrasound. Liver size was calculated using longitudinal dimensions (mm) of right lobe of liver versus height and age.

Mobility assessment. Progressive loss of the ability to ambulate either by crawling (late infantile) or walking (juvenile) is a hallmark of Type II GM1 patients. Due to significant cognitive

medRxiv preprint doi: https://doi.org/10.1101/2024.01.04.24300778; this version posted January 4, 2024. The copyright holder for this preprint (which was not certified by peer review) is the author/funder, who has granted medRxiv a license to display the preprint in perpetuity.
This article is a US Government work. It is not subject to copyright under 17 USC 105 and is also made available for use under a CC0 license.

impairment in this population preventing cooperation with verbal instruction, existing scales of mobility were deemed inappropriate. Therefore, an upright mobility scale (juvenile onset only) and a floor mobility scale (late infantile only) were developed and used in this study. Each is an ordinal scale from 1 (patient unable to perform the skill) to 5 (normal mobility for age). The scales are printed in **Supplementary Table S8**.

Beta-galactosidase enzyme assay. The activity of β-galactosidase was measured using a synthetic fluorogenic substrate as previously described.[3] CSF samples were analyzed for βgal activity using 30μL of sample and serum was analyzed using 10μL of sample. For both CSF and serum, 100μL of substrate was added followed by incubation at 37°C for 1 hour. Protein concentration was determined by Lowry method, utilizing 30μL CSF or 1μL serum, and used to normalize βgal activity to nmol 4MU cleaved/mg protein/hour. Data is expressed as fold of normal and determined by dividing the specific activity of the sample by the average of specific activities from age-matched control samples. Floor effects in β-galactosidase prevent significant declines over time, and so when patients had multiple serum and/or CSF samples, their median value was used.

Electroencephalogram. A 21-channel digital EEG with time locked video and single-lead EKG was performed. EEG electrodes were placed according to the international 10-20 system of electrode placement. The EEG was reviewed by a board-certified pediatric epileptologist (blinded to patient group assignment) using the longitudinal bipolar montage (and when appropriate, referential and transverse montages). Intermittent photic stimulation and hyperventilation were performed in studies that were <1 hour in duration. The total duration of recording ranged from 20 minutes to 12 hours.

A small number of patients also underwent polysomnography (PSG). A one-night, clinical PSG was conducted either on the pediatric ward with a portable system or in the sleep lab at Clinical Center at NIH. Each study included overnight monitoring of

medRxiv preprint doi: https://doi.org/10.1101/2024.01.04.24300778; this version posted January 4, 2024. The copyright holder for this preprint (which was not certified by peer review) is the author/funder, who has granted medRxiv a license to display the preprint in perpetuity. This article is a US Government work. It is not subject to copyright under 17 USC 105 and is also made available for use under a CC0 license.

electroencephalogram, electro-oculogram, ECG, chin and anterior tibial electromyogram, nasal pressure transducer, oral thermistor, a snore sensor, respiratory inductive plethysmography, pulse oximetry and continuous video monitoring. Studies were attended by a certified sleep technologist experienced in pediatrics. Studies were manually scored according to the American Academy of Sleep Medicine Manual version 3, for the scoring of sleep and associated events. The study reports were assessed and approved by a pediatric sleep physician. Given the small number of participants, these results are found in **Supplementary Table S7**.

Magnetic resonance imaging. Brain MRI studies conducted at the NIH Clinical Center were all performed on the same 3T Philips scanner, using an 8-channel SENSE (sensitivity encoding) head coil. Deep sedation with monitored anesthesia care or general anesthesia was used for all participants. Clinical MRI examination included sagittal T1-weighted, axial T2-weighted, coronal STIR, axial fluid attenuated inversion recovery (FLAIR), diffusion tensor, and 3D MP-RAGE (magnetization-prepared rapid acquisition with gradient echo) images, without intravenous contrast. For a subset of participants, MRI studies were obtained from the subjects' referring institution for comparison. All MRI scans were reviewed by the same pediatric neuroradiologist (GV).

Qualitative ratings were made based on the following information. The size of the brainstem was assessed on the mid sagittal T1-weighted images, assessing the surface area of the midbrain, pons and medulla. The cross-sectional area of the corpus callosum was assessed qualitatively on mid sagittal T1-weighted images; for infants, the degree of callosal atrophy was estimated based on age-expected size. The degree of cerebellar atrophy was estimated based on the size of the cerebellar folia. The degree of cortical atrophy was estimated based primarily on the size of the cerebral cortical sulci, also size of the lateral ventricles. For infants, white matter abnormalities were defined primarily as a delay in myelination compared to age expectations, as demyelination/gliosis is not easily assessed in this age group. After infancy,

medRxiv preprint doi: https://doi.org/10.1101/2024.01.04.24300778; this version posted January 4, 2024. The copyright holder for this preprint (which was not certified by peer review) is the author/funder, who has granted medRxiv a license to display the preprint in perpetuity. This article is a US Government work. It is not subject to copyright under 17 USC 105 and is also made available for use under a CC0 license.

when myelination is essentially complete, white matter abnormalities were assessed based on the extent and progression of abnormal increased signal on T2-weighted images. The abnormal increased signal is representative of either demyelination, gliosis or dysmyelination, often in combination. The size and signal intensities of the basal ganglia and of the thalami were also evaluated.

Magnetic resonance spectroscopy. Quantitative single-voxel 1H-MRS was performed on three locations; here we report only on the left centrum semiovale (LCSO). Voxels were graphically prescribed from sagittally-acquired 3D-TFE images reformatted into three planes. 1H-MRS acquisition was performed with PRESS localization, CHESS water suppression, TE=38 ms, TR=2000 ms, and NEX=128. An unsuppressed water spectrum (TR=5000 ms, TE=38 ms, NEX=16) was also acquired for each voxel. Identical gain and shim settings were used for both spectra from each voxel so that metabolite concentrations could be determined. To correct for CSF included within the voxels, we acquired a heavily T2-weighted image with location and slice thickness corresponding to the location of each MRS voxel (FSE; ETL=8; TE=500 ms; TR=3000 ms), and a phantom containing water was placed beside the head and included in the field-of-view of the CSF correction image.

Post-processing of the spectra was performed using LCModel[4], followed by correction for estimated tissue water[5] and T1 of the metabolites within tissue [6]. Because only one echo time was acquired, no correction was made for T2 decay of the metabolites. We analyzed creatine, myo-inositol, choline-containing compounds (“choline”), *N*-acetylaspartate+*N*-acetylaspartyl glutamate (“NAA”), and glutamine+glutamate+gamma-aminobutyric acid (“Glx”). Post-processing for correction of CSF partial volume was done as described in other reports[7-9].

We generated reference curves for each metabolite using data from 38 individuals aged 1-42 years (interquartile range: 3.6–23.3 years). Similarly-acquired and post-processed data from healthy children are not available; instead, children in the reference group were

medRxiv preprint doi: https://doi.org/10.1101/2024.01.04.24300778; this version posted January 4, 2024. The copyright holder for this preprint (which was not certified by peer review) is the author/funder, who has granted medRxiv a license to display the preprint in perpetuity. This article is a US Government work. It is not subject to copyright under 17 USC 105 and is also made available for use under a CC0 license.

asymptomatic or neurologically pre-symptomatic participants in other protocols at our institution who were scanned and post-processed with the same method as the GM1 participants. Adults in the reference group were healthy volunteers. The reference curve for each metabolite was generated by fitting a model of the form $y=Ax^B$ (where x is age and y is metabolite concentration) to the reference data. The difference from the reference curve value was used in all analyses.

Neurodevelopmental Assessments. Speech and language assessments were conducted utilizing informal play and conversation speech tasks involving auditory comprehension and speech production, which were rated by a single SLP on an observational outcome matrix for speech and language functions that also ranged 0 (most severe) to 5 (least severe). Each of these rating scales is reproduced in **Supplementary Table S6**.

Adaptive behavior is the collection of conceptual, social, and practical skills a person uses to function in everyday life. Adaptive behavior is a primary behavioral indicator of the effects of disease progression, and this high degree of clinical relevance is why it was selected for inclusion. The Vineland Adaptive Behavior Scale comprehensive semi-structured parent/caregiver interview is widely used to measure adaptive behavior within the context of neurodevelopmental disorders, and it is appropriate for the full age range (birth to 99 years). Here we began with the version available at study initiation (Vineland-II[10]) and added the current version (Vineland-3[11]) when it became available and its improved psychometric profile was demonstrated.[12] The Vineland was administered by experienced clinicians in a semi-structured interview with caregivers. The Vineland yields an overall composite and domain-level standard scores (mean = 100, SD = 15), and V-scale (mean = 15, SD = 3) and growth scale value (GSV; Vineland-3 only) scores at the subdomain level. GSVs are transformed raw scores that are intended to measure change over time in an individual. GSVs are particularly helpful in

medRxiv preprint doi: https://doi.org/10.1101/2024.01.04.24300778; this version posted January 4, 2024. The copyright holder for this preprint (which was not certified by peer review) is the author/funder, who has granted medRxiv a license to display the preprint in perpetuity. This article is a US Government work. It is not subject to copyright under 17 USC 105 and is also made available for use under a CC0 license.

detecting stability or subtle improvements over time in individuals with significant levels of impairment at baseline.[13,14]

Statistical models for longitudinal data

For outcomes with sufficient numbers of repeated measures, formal longitudinal modeling was attempted, using the following methods. The timescale of interest in this study was chronological age, which was decomposed into between-subject (average chronological age during the study period) and within-subject (duration of participation) effects. While the between-subject effect is useful to understand the developmental course, the within-subject effect more directly corresponds to change that can be expected during a time period relevant to clinical trials (e.g., yearly change). Uncorrelated random effects were used to account for repeated measures within individual (subject-level intercept and slope of within-subject time). While participants were also technically clustered within site and family, the size of the datasets did not support the complexity of modeling this clustering and it was ignored. The parameters of interest from the growth models were the intercept and slope estimates with 95% confidence intervals. Model assumptions were evaluated graphically.

SUPPLEMENTARY RESULTS

Medication use. Participants continued routine clinical care at their home institutions, and medication compliance and its effect on disease was not measured. At their initial visit, eleven (65%) late infantile patients and seven (29%) juvenile patients reported treatment for seizures. There are no FDA-approved medications for the treatment of GM1 gangliosidosis, however off-label use of substrate inhibitor, miglustat, and amino-acid analog, *N*-acetyl leucine are common in GM1 and GM2 patients. During study participation, nine (38%) juvenile and four (24%) late infantile patients took miglustat, and two (8%) juvenile and two (12%) late infantile took *N*-acetyl leucine.

medRxiv preprint doi: https://doi.org/10.1101/2024.01.04.24300778; this version posted January 4, 2024. The copyright holder for this preprint (which was not certified by peer review) is the author/funder, who has granted medRxiv a license to display the preprint in perpetuity. This article is a US Government work. It is not subject to copyright under 17 USC 105 and is also made available for use under a CC0 license.

Growth. Head circumference was within the normal range at the first available assessment, the median [IQR] age- and sex-based percentile was 58 [33.3, 89] in the late infantile group and 40.5 [6.3, 85] in the juvenile group. BMI was also generally within the normal range at the first available assessment. The median [IQR] age- and sex-based Z-score was -0.11 [-0.85, 0.75] in the late infantile group and 0.11 [-1.10, 0.59] in the juvenile group (**Supplementary Figure S8**).

medRxiv preprint doi: https://doi.org/10.1101/2024.01.04.24300778; this version posted January 4, 2024. The copyright holder for this preprint (which was not certified by peer review) is the author/funder, who has granted medRxiv a license to display the preprint in perpetuity.
This article is a US Government work. It is not subject to copyright under 17 USC 105 and is also made available for use under a CC0 license.

Supplementary References

1. Holliday MA, Kim HJ, Zalewski CK, et al. Audiovestibular Characteristics of Small Cochleovestibular Schwannomas in Neurofibromatosis Type 2. *Otolaryngol Head Neck Surg*. Jul 2014;151(1):117-24. doi:10.1177/0194599814529081
2. Dungan S, Gregorio D, Abrahams T, et al. Comparative validity of the American Speech-Language-Hearing Association's national outcomes measurement system, functional oral intake scale, and G-codes to mann assessment of swallowing ability scores for dysphagia. *American Journal of Speech-Language Pathology*. 2019;28(2):424-429.
3. Rosenbek JC, Robbins JA, Roecker EB, Coyle JL, Wood JL. A penetration-aspiration scale. *Dysphagia*. 1996;11:93-98.
4. Provencher SW. Estimation of metabolite concentrations from localized in vivo proton NMR spectra. *Magnetic resonance in medicine*. 1993;30(6):672-679.
5. Ernst T, Kreis R, Ross B. Absolute quantitation of water and metabolites in the human brain. I. Compartments and water. *Journal of magnetic resonance, Series B*. 1993;102(1):1-8.
6. Srinivasan R, Sailasuta N, Hurd R, Nelson S, Pelletier D. Evidence of elevated glutamate in multiple sclerosis using magnetic resonance spectroscopy at 3 T. *Brain*. 2005;128(5):1016-1025.
7. Baker EH, Levin SW, Zhang Z, Mukherjee AB. Evaluation of disease progression in INCL by MR spectroscopy. *Annals of clinical and translational neurology*. 2015;2(8):797-809.
8. Baker EH, Basso G, Barker PB, Smith MA, Bonekamp D, Horská A. Regional apparent metabolite concentrations in young adult brain measured by 1H MR spectroscopy at 3 Tesla. *Journal of Magnetic Resonance Imaging: An Official Journal of the International Society for Magnetic Resonance in Medicine*. 2008;27(3):489-499.
9. Horska A, Calhoun V, Bradshaw D, Barker P. Rapid method for correction of CSF partial volume in quantitative proton MR spectroscopic imaging. *Magnetic Resonance in Medicine: An Official Journal of the International Society for Magnetic Resonance in Medicine*. 2002;48(3):555-558.
10. Sparrow SS, Cicchetti DV, Balla DA. *Vineland Adaptive Behavior Scales, Second Edition.* AGS Publishing; 2005.
11. Sparrow SS, Cicchetti DV, Saulnier CA. *Vineland-3: Vineland adaptive behavior scales*. PsychCorp; 2016.
12. Farmer C, Adedipe D, Bal V, Chlebowski C, Thurm A. Concordance of the Vineland Adaptive Behavior Scales, second and third editions. *Journal of Intellectual Disability Research*. 2020;64(1):18-26.
13. Farmer C, A.J. K, Berry-Kravis E, Thurm A. Psychometric perspectives on developmental outcome and endpoint selection in treatment trials for genetic conditions associated with neurodevelopmental disorder. In: Esbensen A, Schworer E, eds. *International Review of Research in Developmental Disabilities*. Elsevier Inc. Academic Press; 2022:1-39.
14. Farmer C, Thurm A, Troy JD, Kaat AJ. Comparing ability and norm-referenced scores as clinical trial outcomes for neurodevelopmental disabilities: a simulation study. *Journal of Neurodevelopmental Disorders*. 2023;15(1):4. doi:10.1186/s11689-022-09474-6

SUPPLEMENTARY TABLES & FIGURES

Table S1. Mutation classifications. The table shows the evidence and grading scheme used for single nucleotide substitutions. Two deletions (exon 3-5 deletion and exon 5 deletion) not shown in the table are out of frame and thus are pathogenic. Categorizations: Very Strong (PVS), 8 points; Strong (PS), 4 points; Moderate (PM), 2 points; Supporting (PP), 1 point. Classifications: Pathogenic (PATH), 10+ points; Likely Pathogenic (LPATH), 6 to 9 points; Variant of Uncertain Significance (VUS), fewer than 5 points. Cumulative count of classifications: PATH, 28; LPATH, 7; VUS, 0.

See separate file.

Table S2. Audiology Results at Reference Evaluation. Note: Reference evaluation was the most recent, most complete evaluation for each participant. Auditory brainstem response was completed under sedation for 10 (of 15, 67%) of the late infantile onset group and 14 (of 22, 64%) of the juvenile onset group.

	Late Infantile (N=17)	Juvenile (N=24)
Age at evaluation, median [IQR]	4 [3, 6]	12.5 [9, 19]
Peripheral Hearing Sensitivity, n(%)		
Within normal limits	15 (88%)	22 (92%)
Abnormal	2 (12%)	2 (8%)
Auditory Brainstem Response, n(%)		
Within normal limits	8 (47%)	14 (58%)
Abnormal	7 (41%)	8 (33%)
Did not test	2 (12%)	2 (8%)
Behavioral Audiology		
Complete audiogram	0	11 (46%)
Startle response	7 (41%)	3 (13%)
Speech reception or awareness threshold	2 (12%)	8 (33%)
Minimal response level to speech	4 (24%)	0
No behavioral data to report	4 (24%)	2 (8%)

Table S3. Liver enzymes. Data from the earliest available observation per person was used (N = 17 late infantile and N=24 juvenile). The distribution of age in years (median [IQR]) was 4.79 [3.53, 5.68] for the Late Infantile group and 11.82 [7.56, 14.65] for the juvenile onset group.

	Late Infantile (N=17)			Juvenile (N=24)		
	Median [IQR]	*n (%) abnormally high*	ρ_{age}	*Median [IQR]*	*n (%) abnormally high*	ρ_{age}
AST, U/L	73 [44.35, 93.85]	13 (76)	-0.42	33.84 [27.15, 40.47]	7 (29)	-0.53
ALT, U/L	20.8 [16.6, 27]	3 (18)	-0.42	18.7 [16.42, 25]	2 (8)	0.17
GGT, U/L	13.1 [12.11, 26.6]	6 (35)	-0.05	13.8 [8.77, 22.18]	3 (13)	0.1

Table S4a. Summary statistics for cross-sectional MRS data. MRS concentrations (mM) are expressed as deviations from age expectations. Spearman correlations were calculated between difference score and age. The cross-sectional dataset contained the earliest available observation per person. Age at assessment (M[IQR]): Juvenile, 11.66 [7.47, 13.56]; Late infantile, 3.82 [3.43, 5.94].

	**Juvenile (n=21)**		**Late infantile (n=13)**	
	Median [IQR]	ρ_{age}	*Median [IQR]*	ρ_{age}
Creatine difference, mM	0.26 [-0.86, 0.64]	0.22	0.69 [-0.51, 0.85]	-0.57
Myo-inositol difference, mM	1.12 [0.74, 2.18]	0.68	1.6 [0.37, 1.97]	0.28
Choline difference, mM	0.08 [-0.13, 0.17]	-0.31	0.03 [-0.43, 0.23]	-0.46
NAA difference, mM	-2.13 [-4.01, -0.3]	-0.74	-3.45 [-6.81, -0.57]	-0.59
Glx difference, mM	0.21 [-0.05, 1.28]	-0.51	-0.49 [-1.62, 0.17]	-0.15

Table S4b. Juvenile cohort: Parameter estimates and test statistics from mixed effects model of age- and time-related trends in MRS concentration (mM, difference from age expectations). The interpretation of between-subject age is the expected difference in MRS concentration between two people with a difference in average age of participation of 1 year. For example, older participants had higher myo-inositol concentration differences relative to younger participants, at a rate of 0.15 per additional year of age. The interpretation of within-subject time is the expected change within an individual person over the course of 1 year. For example, the average participant had an increase of myoinositol difference of 0.16 mM per year.

	**Between-Subject Age (average age of participation)**			**Within-subject time (time under observation)**		
	Slope (SE)	*95% CI*	*Test statistic*	*Slope (SE)*	*95% CI*	*Test statistic*
Creatine difference, mM	0.03 (0.03)	[-0.03, 0.08]	1.05, p = 0.307	0.08 (0.07)	[-0.06, 0.23]	1.14, p = 0.28
Myo-inositol difference, mM	0.15 (0.06)	[0.04, 0.27]	2.63, p = 0.016	0.16 (0.08)	[0, 0.31]	2.01, p = 0.068
Choline difference, mM	-0.02 (0.01)	[-0.04, 0.01]	-1.16, p = 0.262	0.02 (0.04)	[-0.05, 0.1]	0.61, p = 0.57
NAA difference, mM	-0.27 (0.08)	[-0.42, -0.12]	-3.56, p = 0.002	0.02 (0.1)	[-0.18, 0.21]	0.16, p = 0.872
Glx difference, mM	-0.08 (0.07)	[-0.22, 0.05]	-1.24, p = 0.233	-0.08 (0.18)	[-0.43, 0.26]	-0.48, p = 0.646

Table S5. Subdomain-level V-scale summary statistics for the earliest assessment available for each participant. The first Vineland-II and/or first Vineland-3 assessment available for each person is summarized in this table. Sample sizes and ages were: Late Infantile/Vineland-3: n = 8 (median age: 3.97 [3.06, 5.98]), Late Infantile/Vineland-II: n = 6 (median age: 4.09 [3.46, 4.91]); Juvenile/Vineland-3: n = 14 (median age: 9.59 [7.37, 17.39]), Juvenile/Vineland-II: n = 9 (median age: 17.29 [11.99, 19.38]). Motor excluded as standard scores are not available for all participants. Receptive, Expressive, and Written subdomains belong to Communication; Personal, Domestic, and Community belong to Daily Living; (Interpersonal) Relationships, (Play and) Leisure, and Coping belong to Socialization. Standard scores have a population mean of 100 and SD of 15 (floor = 20), subdomain-level V-scale scores have a population mean of 15 and SD of 3 (floor = 1). IQR = interquartile range (25th – 75th percentile).

		Vineland-3					***Vineland-II***				
Group	Subdomain/ Domain	n	*Standard Score, median [IQR]*	*% ≥2SD below average*	*% at floor*	ρ_{age}	n	*Standard Score, median [IQR]*	*% ≥2SD below average*	*% at floor*	ρ_{age}
Juvenile	Receptive	14	7.5 [1, 11.75]	64	43	-0.74	9	7 [1, 8]	89	44	-0.29
	Expressive	14	1 [1, 8]	79	57	-0.83	9	1 [1, 3]	100	67	-0.81
	Written	13	1 [1, 7]	92	54	-0.67	9	4 [4, 4]	100	22	-0.42
	Personal	14	1 [1, 6]	86	57	-0.63	9	1 [1, 5]	100	56	-0.43
	Domestic	13	6 [1, 8]	77	31	-0.90	9	3 [2, 5]	100	11	-0.53
	Community	13	3 [1, 6]	85	31	-0.81	9	2 [1, 5]	100	44	-0.44
	Relationships	14	7.5 [2.25, 10.75]	64	14	-0.72	9	3 [2, 7]	100	22	-0.62
	Leisure	14	7.5 [1, 11.75]	57	43	-0.81	9	3 [1, 7]	100	33	0.09
	Coping	13	8 [3, 11]	62	23	-0.89	8	5 [4.75, 7.5]	88	0	0.12
	Fine	8	4.5 [1, 9.25]	75	50	-0.93	0				
	Gross	8	4.5 [1, 11]	62	50	-0.91	0				
	**Composite**	14	44 [24.5, 70]	71	21	-0.88	9	29 [26, 53]	100	22	-0.55
	**Communication**	14	36 [20, 68.5]	79	43	-0.80	9	28 [28, 52]	100	0	-0.77
	**Daily Living**	14	45.5 [20, 65.75]	79	36	-0.93	9	28 [25, 51]	100	0	-0.56
	**Socialization**	14	57 [30.25, 76.75]	57	21	-0.84	9	42 [37, 55]	100	11	-0.52
	**Motor**	8	44 [20, 75.5]	62	50	-0.91	0				
Late Infantile	Receptive	8	5 [1, 9.75]	75	50	-0.75	6	5 [5, 5.75]	83	0	-0.03
	Expressive	8	2.5 [1, 5]	88	50	-0.73	6	4 [4, 7]	100	0	0.03
	Written	7	5 [1, 10.5]	71	43	-0.96	6	9.5 [9, 10]	50	0	-0.93
	Personal	8	1.5 [1, 7]	75	50	-0.80	6	5 [4.25, 5.75]	100	0	-0.06
	Domestic	7	9 [5, 12]	57	0	-0.95	6	8.5 [8, 9.75]	67	0	-0.75
	Community	7	7 [2, 11]	57	14	-0.95	6	7.5 [6.25, 9.5]	67	0	-0.77
	Relationships	8	6.5 [2.5, 9.5]	75	25	-0.89	6	7 [4.5, 8.75]	83	0	-0.03
	Leisure	8	7.5 [1, 9]	75	38	-0.81	6	8 [6.25, 9.75]	67	0	-0.64
	Coping	8	8.5 [6.25, 10.5]	62	0	-0.90	6	8 [7.25, 8.75]	100	0	-0.72
	Fine	7	5 [2.5, 8.5]	86	29	-0.70	6	4 [3, 5.75]	100	17	-0.41

Gross	7	1 [1, 6.5]	100	57	-0.38	6	5.5 [4.25, 7.5]	83	0	-0.14
**Composite**	8	56.5 [32, 65.25]	75	0	-0.86	6	43 [39.25, 54.25]	100	0	-0.60
**Communication**	8	43 [20, 65]	75	38	-0.85	6	43 [42, 51.5]	100	0	-0.49
**Daily Living**	8	56.5 [38, 68.5]	75	0	-0.73	6	46 [43.75, 59.5]	100	0	-0.61
**Socialization**	8	60 [35.25, 69.5]	75	0	-0.80	6	56 [48.25, 66.75]	100	0	-0.14
**Motor**	7	36 [20, 64]	71	43	-0.41	6	38.5 [31.75, 51.25]	100	0	-0.26

Table S6. Scoring scales used for outcome measures of speech, language, aspiration/laryngeal penetration, swallowing, and dietary restrictions, partially adapted from ASHA/NOMs and Rosenbek scales.

Domain	Rating	Description
Speech	5	Normal age-appropriate Speech/Language Skills
	4	Mild impairment of speech clarity, rate, rhythm, misarticulations
	3	Moderate impairment of rate rhythm clarity, dysarthria
	2	Severe impairment of speech absent, non-intelligible
	1	Profound Speech-non intelligible or absent speech
	0	Intermittent speech vocalizations
Language/Communication	5	Language Skills are within Age Range
	4	At least one area is deficient: sentence length/complexity; vocabulary; morphology use of language pragmatics
	3	At least two of the following areas deficient: sentence length/complexity; syntax; vocabulary; morphology pragmatics
	2	At least three of the following areas deficient; sentence length/complexity; syntax; vocabulary; morphology; pragmatics
	1	At least four of the following areas are deficient sentence length/complexity; syntax; vocabulary; morphology; pragmatics
	0	No Communicative Intent
Aspiration Laryngeal Penetration	5	Contrast Does not Enter Airway; no Signs /Symptoms Aspiration
	4	Risk Minimal Intermittent Laryngeal Penetration with retrograde Excursion on one or more textures; Cough throat clear with one or more textures
	3	Contrast Enters the airway consistently on one or more textures
	2	Aspiration 10-50%; volitional cough
	1	Aspiration of one immediately; Delayed aspiration from retained material; no cough
	0	Aspiration of one or more textures immediately
Swallowing	5	Individual's ability to swallow is normal; Swallowing would be safe and efficient for all consistencies. Compensatory strategies are effectively used when needed
	4	Swallowing is safe and the individual eats and drinks with minimal cueing; Many need to avoid specific food item or require additional time to eat
	3	Individual's swallowing is with minimal diet restrictions and/or occasional requires cueing to use compensatory strategies; all nutrition needs are met by mouth
	2	Swallowing is safe; Moderate cueing to use strategies; moderate diet restrictions/requires supplements or some tube
	1	Alternative method of feeding is required as individual takes less than 50% nutrition and/or hydration by mouth
	0	Individual not able to swallow safely by mouth for nutrition and hydration. Alternative method of feeding
Dietary Restrictions	5	No Restrictions
	4	Minimal Restriction: Diet is one level below a regular diet with a change in liquid and/or solid
	3	Moderate Restriction: Diet is two or more levels below regular status in liquid and or solid textures but not both
	2	Maximal restriction: Diet is two or move levels below regular status in solid and/or liquid
Solids	5	No restrictions
	4	Reduced one level meats are soft

	3	Reduced two levels meats are chopped or ground; Mechanical soft
	2	Reduced three levels puree diet
Liquids	5	No restrictions
	4	Mild: Nectar thick
	3	Moderate: Honey thick
	2	Severe: Pudding thick

Table S7. Polysomnography results for seven participants. LI = late infantile, JUV = juvenile, BMI = body mass index, AHI = apnea-hypopnea index, SWS = slow wave sleep, REM = rapid eye movement, WASO = wake after sleep onset

Group	Sex	Age Range (years)	BMI	Pedi-Epworth Score	Recording Time (mins)	Total Sleep Time (mins)	Sleep Efficiency	Overall AHI (per hr)	Sleep Onset Latency (mins)	N2 Latency (mins)	SWS Time (mins)	REM Latency (mins)	REM Time (mins)	REM % of TST	WASO (mins)
LI	Male	5 – 9	16.2	14	606	358	59.20%	0.5	125	129	0	171	51	14.20%	120
LI	Male	5 – 9	16.1	11	649	487	75.10%	1.5	5	8	0	179	61	12.50%	156
LI	Female	0 – 4	18.5	4	514	451	87.70%	0.3	20	23	227.5	95	87	19.30%	43.5
JUV	Male	30 – 34	19.8	5	558	335	60.10%	0.9	93	103	0	179	17	5.10%	128.5
JUV	Female	20 – 24	23.7	0	437	41	9.40%	1.5	217	220	0	0	0	0%	173.5
JUV	Female	15 – 19	17.4	6	666	569	85.40%	0.5	3	11	0	64	180.5	31.70%	94
JUV	Male	5 – 9	16.4	3	522	468	89.80%	1	0	0	120.5	205	84	17.90%	52.5

Table S8. Rating anchors for the mobility assessment. The floor mobility scale was used for the late infantile group and the upright mobility scale was used for the juvenile onset group.

Rating	Floor Mobility	Upright Mobility
5	Crawls in 4-point independently	Ambulation is normal for age
4	Scoots independently when sitting	Independent ambulation, may be unsteady
3	Sits without support	Ambulates independently with mobility aid due to unsteady gait
2	Sits with support	Ambulates only with the assistance of another person (with or without a mobility aid) due to unsteady gait
1	Rolls independently – rolling belly to back	Able to pull to stand, unable to ambulate
0	Unable to roll	Non-ambulatory, dependent for wheeled mobility (WC or stroller)

Figure S1. Parent-reported acquisition and loss of major milestones. Parent report of acquisition and loss of milestones was obtained at the last contact (for most participants, this was August 2023, see **Figure** 1). For Late Infantile, the age when these data were obtained/verified ranged 2.7 – 22.0 years, (median = 6.1, IQR = 4.2 – 11.2); for Juvenile 3.8 – 35.5 years, (median = 21.1, IQR = 14.2 – 26.1). Age of acquisition was coded as "on time" or "delayed" according to WHO or CDC normative data (thresholds: sitting: 8 months; cruising: 14 months; walking: 16 months; words = 12 months). If the participant acquired a skill, loss was queried. The number of participants in each category is inset in black text, and in panel B, the age range of those who had not yet lost the skill is inset in white text.

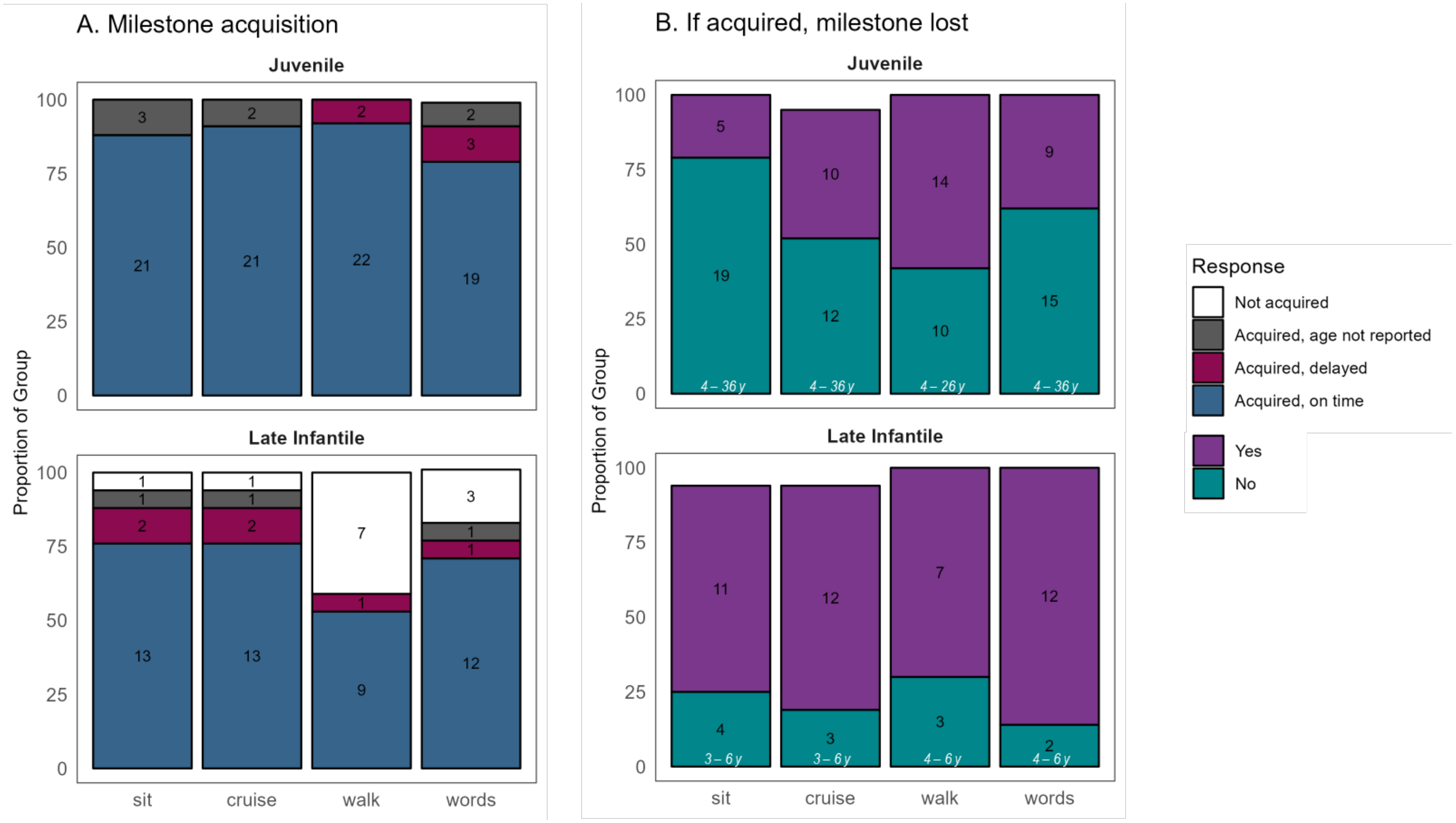

Figure S2. Floor (late infantile, red) and upright (juvenile, black) mobility scale ratings at baseline (Panel A) and across repeated measures (Panel B). Note that the qualitative ratings made on a scale of 0 (most impaired) to 5 (normal) differ between late infantile participants (red points; floor mobility) and juvenile participants (black points; upright mobility). See supplementary tables for qualitative rating descriptions. Slight random jitter added to Y-axis of cross-sectional panel (panel A) to reduce overplotting.

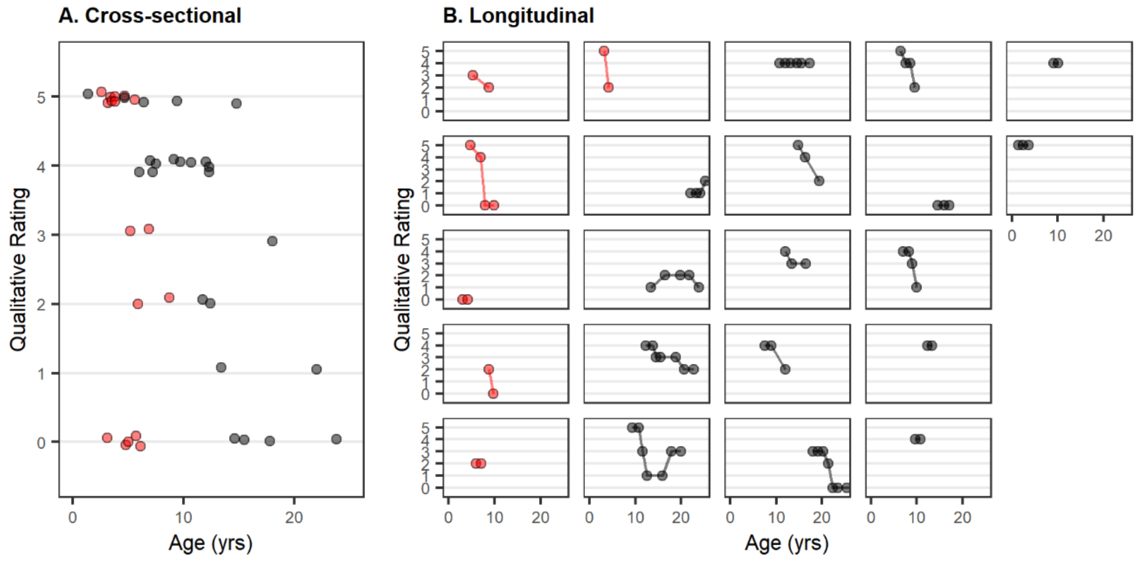

Figure S3. β-galactosidase enzyme activity in CSF and serum. Panel A: Fold-change from pediatric control sample. Panel B: Related to lack of variability in CSF and serum values, no correlation between CSF and serum was observed (Late infantile: ρ = 0.2; Juvenile: ρ = .04). Dotted line indicates 1:1 correlation. Both panels: red = late infantile, black = juvenile; median b-gal value per person.

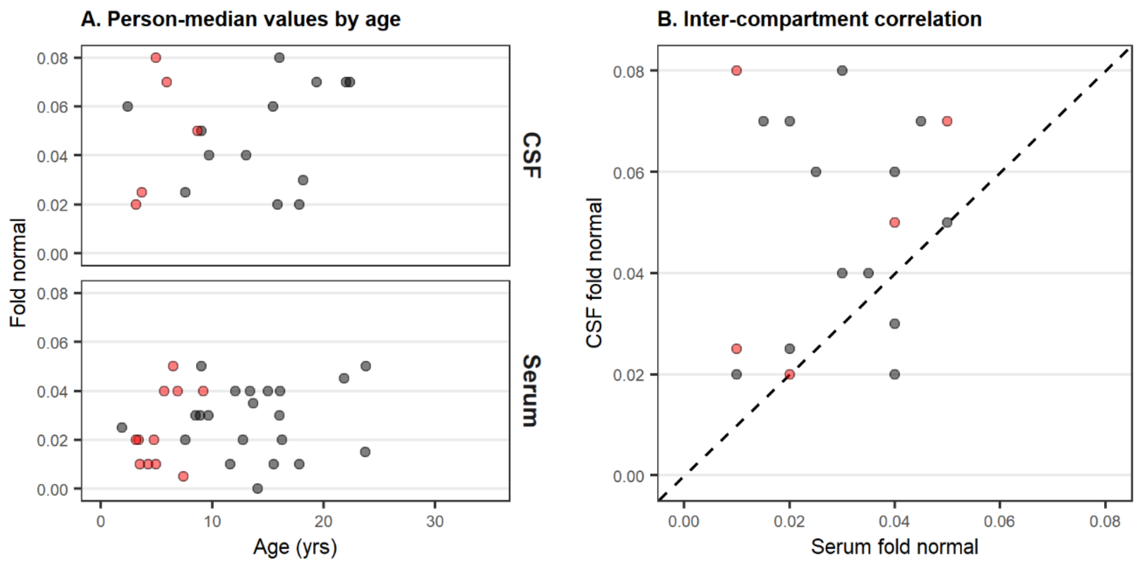

S4. Longitudinal data on liver enzymes for late infantile (red) and juvenile (black) onset groups. Available data for each participant are plotted against their age and connected by a solid line. Concentrations were compared to reference range values and expressed as fold change from the upper limit of the reference range (dotted reference line at 1 indicates the upper limit of the reference range). Lines connect observations from a single person.

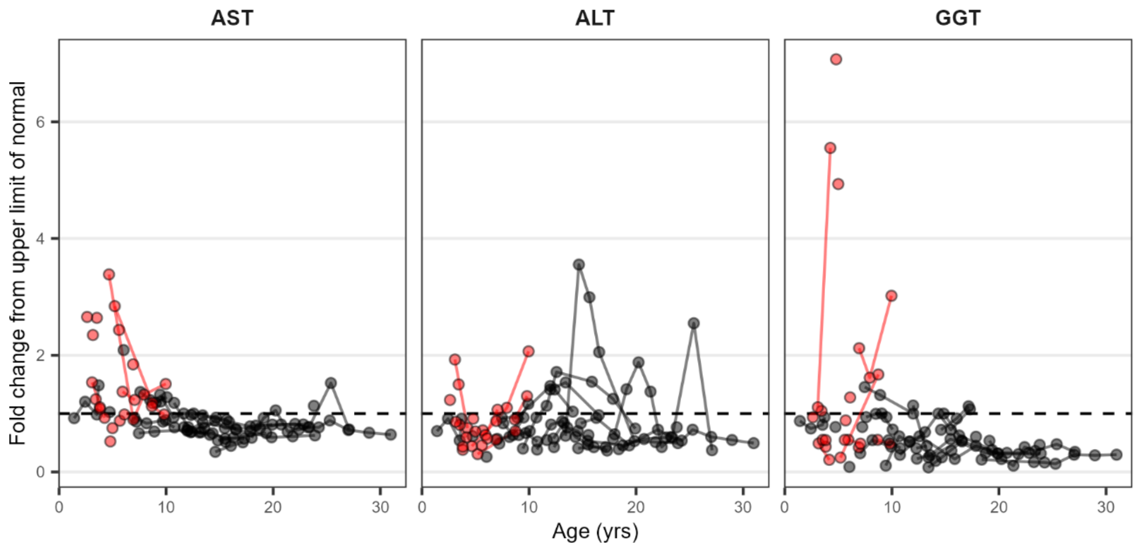

Figure S5. Longitudinal data showing age progression of metabolite concentration (mM) in LCSO relative to normative expectations. NAA: N-acetyl aspartic acid. Glx: Glu+Gln+GABA. Red = late infantile onset; black = juvenile onset. Lines connect observations from the same participant. Points show difference in concentration (mM) from age expectation, which is illustrated by the dotted line at zero.

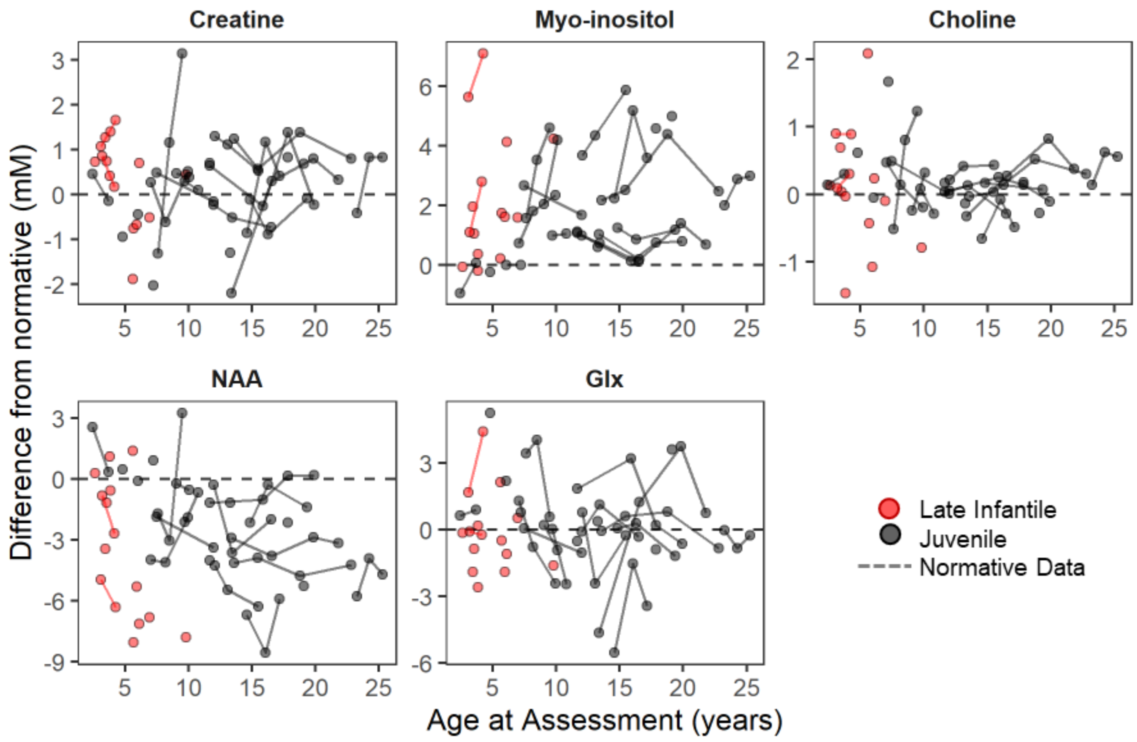

Figure S6a. Speech qualitative ratings at first observation (panel A) and across longitudinal observation (panel B) for late infantile (red) and juvenile (black) groups. Qualitative ratings made on a scale of 0 (most impaired) to 5 (normal for age); see supplementary table for full description. Slight random jitter added to Y-axis of cross-sectional panel (panel A) to reduce overplotting.

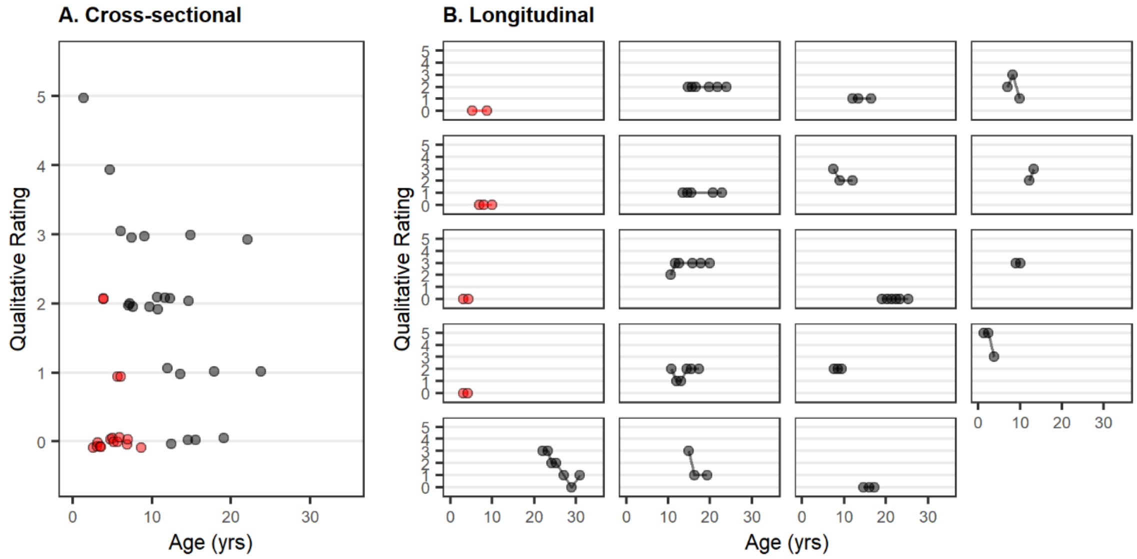

Figure S6b. Language qualitative ratings at first observation (panel A) and across longitudinal observation (panel B) for late infantile (red) and juvenile (black) groups. Qualitative ratings made on a scale of 0 (most impaired) to 5 (normal for age); see supplementary table for full description. Slight random jitter added to Y-axis of cross-sectional panel (panel A) to reduce overplotting.

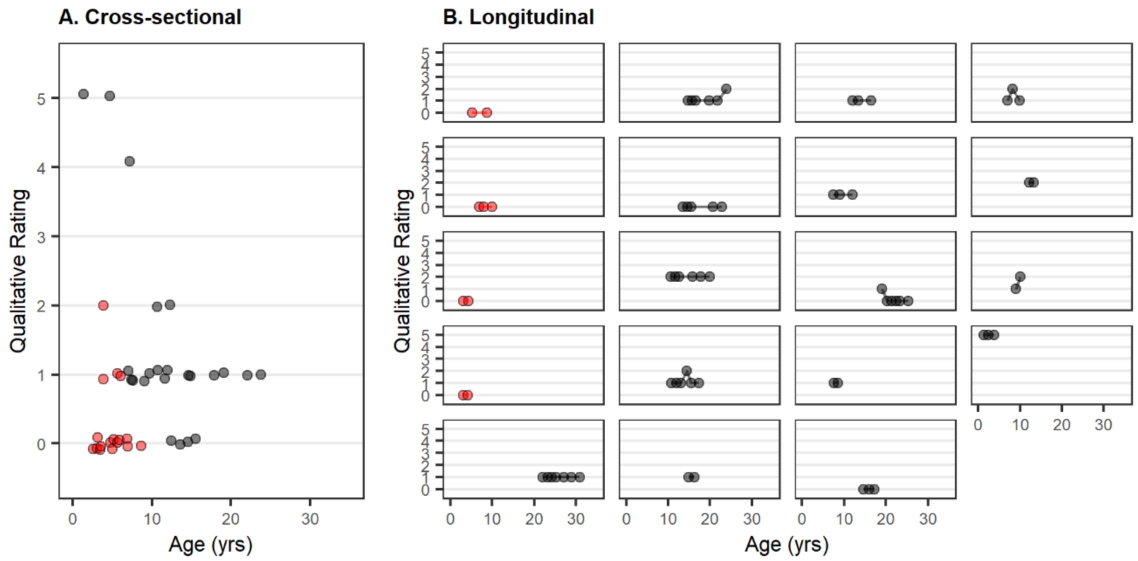

Figure S7a. Longitudinal trends in Vineland domain-level standard scores for Late Infantile (left) and Juvenile onset (right). For each participant, the form (Vineland-II or Vineland-3) with a longer follow-up period was selected, so that each participant appears only once per panel. Where Vineland-II scores were used, the markers are black, and the markers are gray for Vineland-3. Solid lines connect observations from the same participant, but different participants are not otherwise demarcated. Dotted lines indicate normative expectations (population mean = 100, SD = 15).

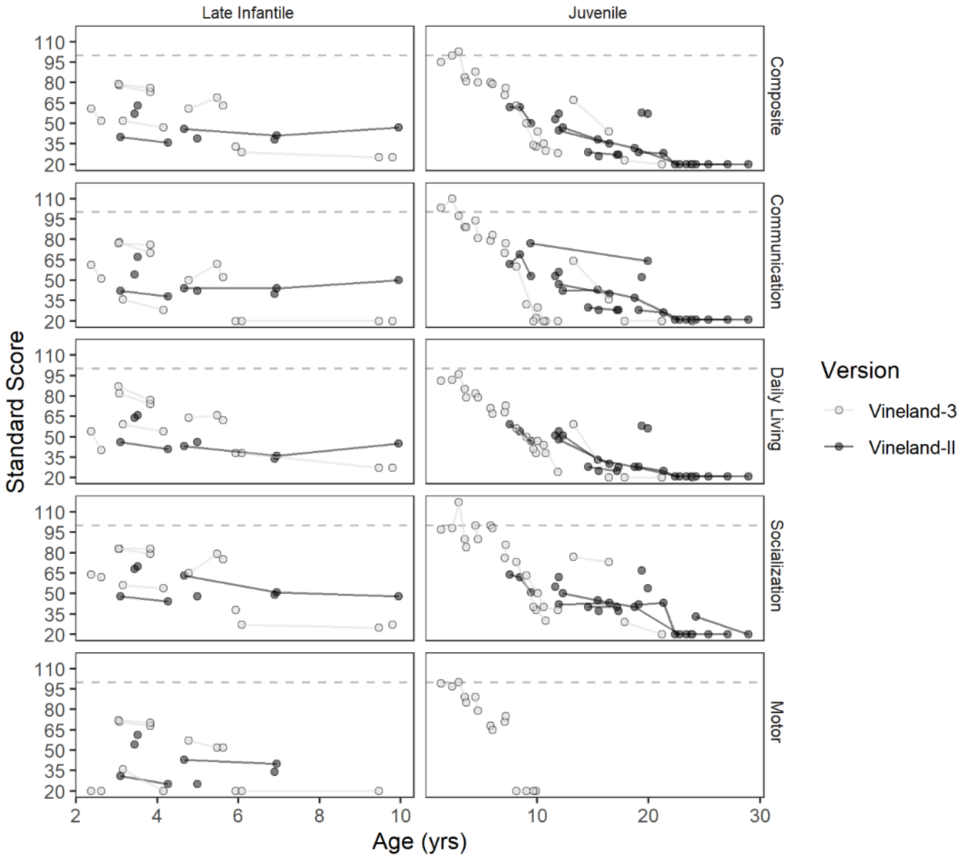

Figure S7b. Longitudinal trends in Vineland subdomain-level V-scale scores for Late Infantile (left) and Juvenile onset (right). For each participant, the form (Vineland-II or Vineland-3) with a longer follow-up period was selected, so that each participant appears only once per panel. Where Vineland-II scores were used, the markers are black, and the markers are gray for Vineland-3. Solid lines connect observations from the same participant, but different participants are not otherwise demarcated. Dotted lines indicate normative expectations (population mean = 15, SD = 3).

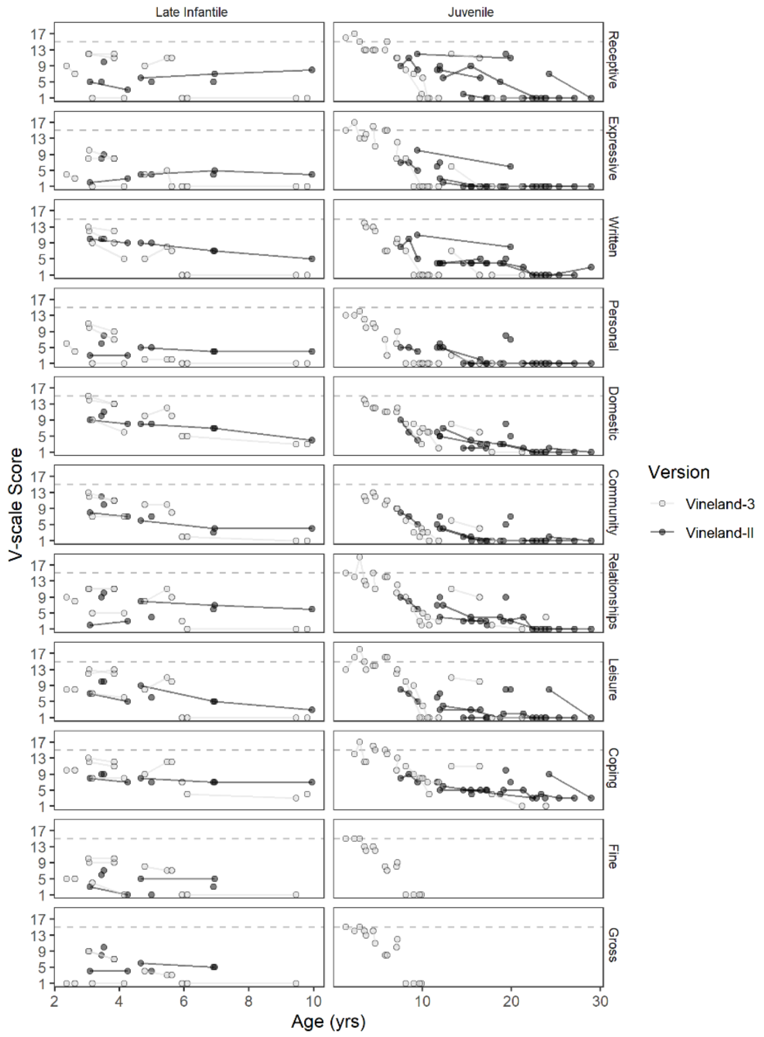

Figure S7c. Longitudinal trends in Vineland subdomain-level growth scale values (GSVs) for Late Infantile (left) and Juvenile onset (right). Unlike the preceding plots 3a and 3b, all available Vineland-3 data are plotted. Solid lines connect observations from the same participant, but different participants are not otherwise demarcated. Dotted lines indicate normative expectations (median value per age equivalent).

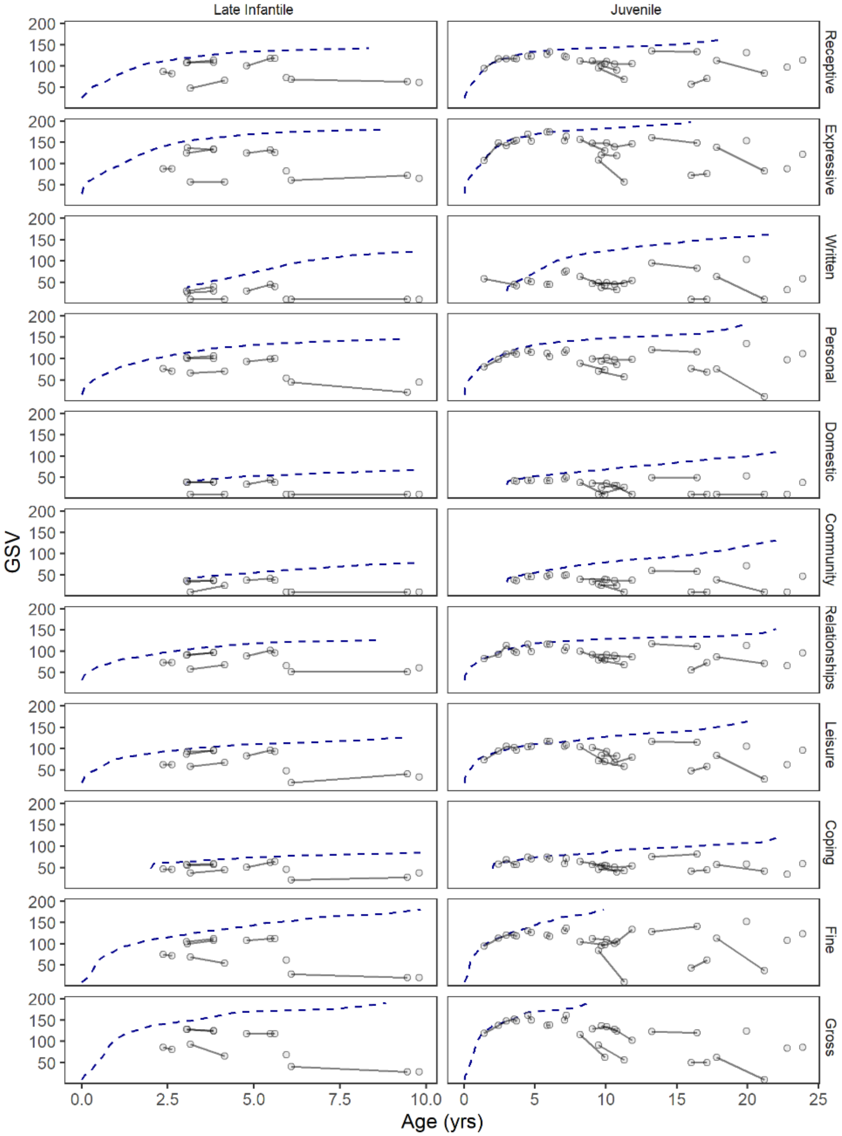

Figure S8. Age- and sex-based BMI Z-scores at the first assessment. Panel A: BMI Z-scores plotted by age. Panel B: BMI Z-scores plotted by group. Both panels: Red = late infantile onset, black = juvenile onset. CDC normative data for age 2 – 20 years were used; Z-score was not calculated for one participant aged 1.5 years and for two participants aged >20 years the 20-year norms were used.

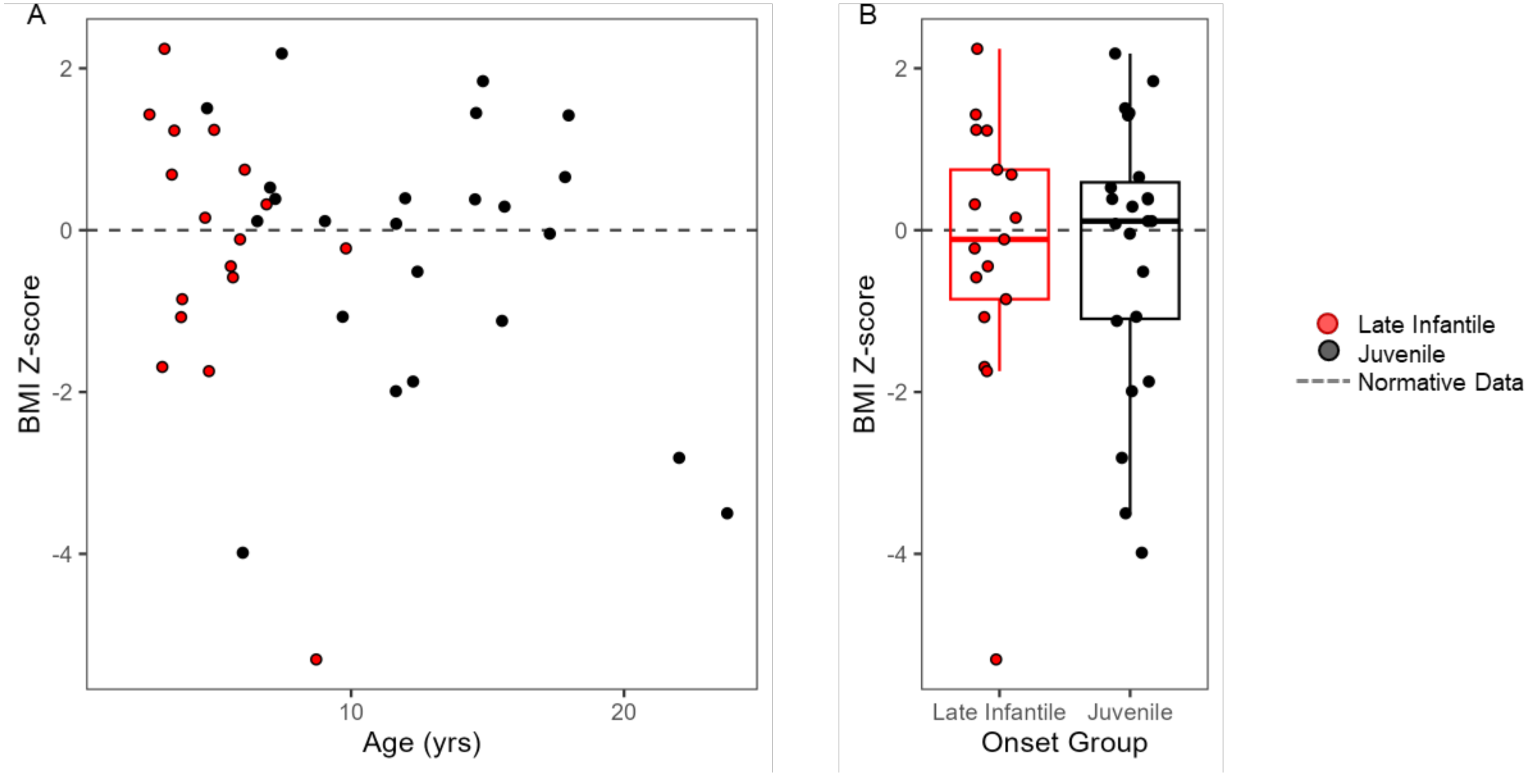

Supplement: Supplement 2 [file NIHPP2024.01.04.24300778v1-supplement-2.pdf]
